# High throughput screening and evaluation of salt-tolerant mutants from an EMS collection of *Cucurbita pepo*

**DOI:** 10.3389/fpls.2025.1548576

**Published:** 2025-05-15

**Authors:** Sonsoles Alonso, Keshav Gautam, Jessica Iglesias-Moya, María Segura, María del Mar Rebolloso, María del Carmen Salas-Sanjuán, Cecilia Martínez, Manuel Jamilena

**Affiliations:** ^1^ Department of Biology and Geology, Agrifood Campus of International Excellence (CeiA3), and Research Center CIAMBITAL, University of Almería, Almería, Spain; ^2^ Department of Agronomy, and CIAIMBITAL Research Center. University of Almería, Almería, Spain

**Keywords:** *Cucurbita pepo*, screening, salt stress, germination, EMS, mutants, salt tolerance, rootstocks

## Abstract

Salinity is a major environmental stress limiting crop growth and yield in many arid and semi-arid regions of the world. In this study, we used a germination-based selection method to screen an EMS collection of *Cucurbita pepo*, consisting of 3,751 M_2_ lines, for salt tolerance. The screening resulted in the identification of six salt-tolerant mutants that exhibited enhanced germination and plant growth under salt stress conditions. This enhanced salt tolerance was found to be associated with increased production of proline, soluble sugars, and anthocyanins, which are known to exert osmoprotective and ROS scavenging functions. The mutant lines TS-1378 and TS-2075 were selected to be tested as rootstocks and showed a positive effect on the vegetative growth of scions under standard and saline conditions. In conclusion, the research provides an efficient protocol for high throughput screening for salt tolerance, novel useful mutants to study the mechanism behind salt tolerance, and valuable genetic resources for pumpkin breeding.

## Introduction

Salinity is an important environmental stress limiting crop growth and productivity in many arid and semi-arid regions of the world, affecting up to 20% of arable land and up to 50% of irrigated land (Ulas et al., 2020). Natural and anthropogenic activities have led to an increase in salt-prone areas, and it is predicted that half of the current arable land could be lost by 2050 (Jamil et al., 2011). Crops growing under saline conditions exhibit a variety of problems, including shorter life cycles, reduced growth, metabolic disorders, ion toxicity, drought, and reduced nutrient uptake and photosynthetic activity, which can lead to reduced productivity or even death (Flowers, 1999; Lutts et al., 1999; Zhu, 2002; Wang et al., 2003). Therefore, breeding for salt-tolerant cultivars is becoming a necessity to utilize saline lands and a challenge for the scientific community today.

Salinity stress affects plant growth in two main ways. High soil salinity limits the ability of roots to absorb water (osmotic stress), followed by ionic toxicity of Na^+^ and Cl^-^ ions (Isayenkov and Maathuis, 2019). To cope with salinity, plants have implemented various mechanisms that improve tolerance to the associated stress, including changes in morphology, anatomy, water relations, photosynthesis, hormonal profile, toxic ion distribution, and biochemical adaptation (Hernández et al., 2001; Parida and Das, 2005; Ashraf and Harris, 2013; Acosta-Motos et al., 2015a, 2015b). In general, a larger root system appears to be better for plants because it allows them to penetrate deeper soil layers to obtain water and nutrients (Franco et al., 2011). In addition, increased development of the main and lateral roots is associated with greater robustness and accumulation of reserves and, presumably, greater tolerance to salt stress (Cameron et al., 2006; Franco et al., 2011; Gómez-Bellot et al., 2013). However, other studies have shown that species with small roots may be more advantageous for shoot development (Ma et al., 2010). Additionally, a reduction in leaf area can minimize water loss by transpiration due to a lower number of stomata, which is an avoidance strategy (Savé et al., 1994; Ruiz-Sánchez et al., 2000). This effect can favor the retention of toxic ions in the roots, limiting their accumulation in the aerial parts of the plant (Colmer et al., 2005; Munns and Tester, 2008). This results in an increased root-to-shoot ratio, which is thought to improve the source/sink ratio for water and nutrients under saline conditions (Zekri and Parsons, 1989).

In addition to plant size, another interesting strategy to cope with salinity is a biochemical adaptation that leads to the production of different types of compounds. Osmolytes, such as proline or sugars, help restore homeostasis, maintain cell volume and turgor, and reduce oxidative damage by protecting membranes and proteins. Antioxidant metabolism aims to control damage caused by reactive oxygen species (ROS). This metabolism involves two strategies, the activation of antioxidant enzymes such as catalase (CAT, EC 1.11.1.6) or superoxide dismutase (SOD, EC 1.15.1.1), among many others (Kav et al., 2004; Chen et al., 2009; Sugimoto and Takeda, 2009), as well as the activation of non-enzymatic, which includes the synthesis of molecules such as carotenoids, flavonoids (such as anthocyanins), as well as reduced glutathione and ascorbic acid (Ahmad et al., 2009).

Zucchini (*Cucurbita pepo* L.) is an important horticultural crop, grown in arid and semi-arid regions. Some genotypes of the *Cucurbita* genus exhibit salt tolerance and can therefore be used as rootstocks to improve the growth and yield of some horticultural crops under salt stress (Ulas et al., 2020). Grafting onto suitable rootstocks is an important technique to facilitate the adaptation of important crops to soil challenges, including biotic and abiotic stresses. This technique is widely used for the cultivation of some important cucurbits and solanaceous species (Pogonyi et al., 2005; Schwarz et al., 2010). Therefore, the identification of genotypes capable of improving salt tolerance is one of the necessary objectives for cucurbit breeding. In this sense, the identification of novel and valuable mutations is a widely used strategy, and mutation-derived varieties have been released in many important crops such as rice, wheat, cotton, sesame, grapefruit, and banana (Ahloowalia et al., 2004). Among the mutagenesis methods, the chemical agent ethyl methanesulfonate (EMS) is widely used because it is a simple method that provides a very high mutation frequency (Greene et al., 2003; Dahmani-Mardas et al., 2010; Okabe et al., 2011; Vicente-Dólera et al., 2014; García et al., 2018). Salt-tolerant EMS mutants have been identified in Arabidopsis (Quesada et al., 2000), rice (Takagi et al., 2015), *Cucurbita moschata* (Chen et al., 2018) or wheat (Lethin et al., 2020). However, to our knowledge, no salt-tolerant EMS mutant of *C. pepo* has been reported.

Given the complexity of the genetic network controlling salinity tolerance, the identification of tolerant mutants is a challenging task to gain insight into the trait. In addition, tolerance may be restricted to one developmental stage or may involve multiple stages of plant development, making selection more complex. Although germination is recognized as one of the most sensitive stages of the plant cycle to salinity (Munns and Tester, 2008; García-Hernandez et al., 2018; Zörb et al., 2019), most studies have focused on seedling or mature plant stages rather than seeds. The root bending assay (Wu et al., 1996), salt injury index (Mujtaba et al., 2018), crop yield (Stratena et al., 2019), or plant biomass (Takagi et al., 2015; Chen et al., 2018) are some methods that have been used to evaluate plant salt tolerance.

In this study, we propose a rapid and efficient germination stage-based method for a high-throughput screening of 3,751 M_2_ lines from an EMS squash collection. The inheritance of the salinity tolerance phenotype was evaluated up to the M_4_ generation, and six different mutant lines were identified as genetically stable. The salt-tolerant phenotype of the mutant lines was later confirmed at the seedling and plant stages and was found to be associated with greater production of osmoprotectants in leaves and roots compared. In addition, the potential of the two best mutant genotypes as rootstocks for zucchini scions was analyzed. The identified *C. pepo* salt-tolerant mutants may be used not only to enrich germplasm resources for breeding salt tolerance, but also as research materials to identify salt-tolerance-related genes and to better understand the salt-tolerance mechanism in plants.

## Materials and methods

### Plant material

The plant material used for the salinity tolerance screening was an EMS collection consisting of 3,751 M_2_ lines of *C. pepo*. A stepwise methodology was followed to identify and characterize the tolerant phenotype. First, the salt-tolerant phenotype was detected at the germination stage in segregating M_2_ populations (where the phenotype was expected to segregate). The inheritance of the phenotype was then assessed in subsequent M_3_ and M_4_ generations (where the phenotype was expected to be genetically stable). This was done by self-pollinating both tolerant and non-tolerant seeds from each candidate M_2_ line to obtain the M_3_ generation. The same process was repeated in M_3_ to produce the M_4_ generation.

The collection’s salt-sensitive genetic background, MUCU16, was used as the WT reference phenotype in all experiments. To avoid the effect of seed age and storage conditions in the germination test, MUCU16 seeds were obtained in parallel with the M_3_ and M_4_ mutant generations. Mutant and MUCU16 plants were transplanted and grown in the same greenhouse under standard conditions. After pollination, fruits were allowed to grow and mature for 60 days before seeds were collected, cleaned and stored under the same conditions. Seeds were extracted, sterilized with 10% bleach, and then dried and stored in the dark at 6°C for 3–6 months. After this storage period, the mutant and MUCU16 lines were used to evaluate germination capacity and plant growth.

### Salt tolerance screening at the germination stage

To select an appropriate salt concentration for screening, a dose-response curve was constructed using the MUCU16 genetic background. The NaCl concentrations tested were 85, 150, 200, 250 and 300 mM. Germination was evaluated according to the protocol described below, selecting a NaCl concentration at which the genetic background did not germinate at all (**Supplementary Figure S1A**). After determining the appropriate salt concentration (300mM), 3,751 M_2_ lines comprising the mutant collection were screened to select salt-resistant lines. The screening results were validated in M_3_ and M_4_ generations by using a concentration of 200 mM NaCl.

For germination assays, seeds were incubated in 50 mL Falcon tubes containing 25 mL of distilled water (control) or 200/300 mM NaCl for 16 h at 24°C in the dark with continuous shaking. After imbibition, the seeds were sown in Petri dishes between two filter papers moistened in the appropriate solution. The seeds were then incubated in a growth chamber in the dark at 24°C and 80% relative humidity (RH). 60 seeds were used for each genotype and treatment, and germination was assessed every 2 h for 3 days. Seeds were considered germinated when the seed coat was broken, and the primary root emergence was visible (>1 mm).

### Evaluation of salinity tolerance at seedling stage: etiolation assays

The salt-tolerant mutant lines selected at germination were characterized at the seedling stage. Dark etiolation was used as a test to determine the response of the plant to 100mM salt in two independent experiments. A total of 80 seeds per genotype and experiment were incubated in 50 mL Falcon tubes containing 25 mL of distilled water for 16 h at 24°C in the dark with continuous shaking. Seeds were then sown in Petri dishes between two filter papers moistened with distilled water and incubated in a growth chamber in darkness at 24°C and 80% RH for 72 h. After this time, the germinated seeds were sown in 0.5L pots containing vermiculite, randomized between control and saline treatments. Control and saline solutions were prepared with distilled water supplemented with Hoagland’s nutrient solution (1g/L) and pH adjusted to 5.8; in addition, 100 mM NaCl was added to the saline solution. The conductivities of the control and salt solutions were 1.731 and 11.569 dS/m, respectively. The pots containing the germinated seeds were incubated in a growth chamber under the same conditions described for germination. After 72h, the etiolated seedlings were measured after removal from the pot and washing them to remove the vermiculite. The NaCl concentration used for this assay was selected previously after analyzing the effect of different concentrations of NaCl (30, 60, 100 and 150 mM) on the growth of MUCU16 seedlings according to the same protocol described (**Supplementary Figure S1B, C**). For each independent seedling of each genotype and treatment, shoot and parent root length and fresh shoot and root biomass were measured. The percentage of reduction of each parameter in response to salt stress in WT and mutant plants were determined with respect to plants of the same genotype growing under control conditions.

### Assessing the effect of salinity on the vegetative growth of MUCU16 and mutant plants

The effect of salt stress on the vegetative growth of the MUCU16 and M_4_ mutant lines was studied at the plant stage. All individuals of the different genotypes were germinated and developed under standard conditions, in a chamber at 24°C, 60% RH and a photoperiod of 16 h light/8 h dark. They were watered with the control solution until time zero (T0), which was established when the plants had two true leaves. At this time point, plants of each genotype were separated into control and saline (100 mM NaCl) treatments. Several growth parameters, including root length, plant height, root and leaf fresh weight and leaf area were compared between genotypes at 0, 4, 7, 11, and 14 days after treatment under control and stress conditions. Three independent replicates of three plants each were analyzed for each genotype and irrigation condition at each time point.

A growth index was determined for each line and tissue (leaf and root). It was calculated from the following expressions:


$${\text{Growth index}}_{leaf}=(\frac{\text{Plant height under NaCl}}{\text{Plant height under control}}+\;\frac{\text{Aerial biomass under NaCl}}{\text{Aerial biomass under control}}+\;\frac{\text{Leaf area under NaCl}}{\text{Leaf area under control}})/3$$

$${\text{Growth index}}_{root}=(\frac{\text{Root length under NaCl}}{\text{Root length under control}}+\;\frac{\text{Root biomass under NaCl}}{\text{Root biomass under control}})/2$$

### Assessing the effect of salinity on stress-relate metabolites

Metabolites were measured in three replicates of fresh leaves and roots of each genotype and treatment collected at T11 (11 days after treatments). Concentrations of proline, soluble sugars and anthocyanins, malondialdehyde (MDA), and H_2_O_2_ were determined. Proline was determined by the ninhydrin method with minor modifications. 300 mg of fresh sample was incubated in 6 mL of 60% ethanol at 4°C for 12 h. After centrifugation at 14,000 g for 5 min at 4 °C, 0.3 mL of this solution was mixed with 0.6 mL of 1% ninhydrin, dissolved in 60% acetic acid, and incubated at 95°C for 20 min. Proline concentration was finally determined by spectrophotometry at 520 nm and expressed as µmol/g fresh weight (FW). The concentration of soluble sugars was determined by the phenol-sulfur method with minor modifications. 300 mg of fresh sample was incubated in 10 mL of 80% ethanol at 80°C for 1 h, and 1 mL of this solution was then mixed with 1 mL of a 2% phenol solution and 5 mL of 95–97% sulfuric acid. Soluble sugars were determined at 490 nm and expressed as µmol/g FW. Anthocyanin content was determined according to Mancinelli (1990). 100 mg of fresh sample was incubated for 12 h at 4°C in 3 ml of a solution of acidified ethanol with 37% HCl. Spectrophotometric measurements were made at 530 and 657 nm and the concentrations were expressed as µg/g FW. The anthocyanin content was finally determined from the expression A530-0.25*A657.

MDA was determined according to Heath and Packer (1968) with minor modifications. 0.5 g of fresh sample was mixed with 200 µL of 4% BHT dissolved in ethanol and 1 mL of 20% TCA. After homogenizing the mixture, it was centrifuged at 12,000 g for 10 min at 4 °C. 250 µL of the supernatant was mixed with 750 µL of 0.5% TBA in 20% TCA, and incubated at 94 °C for 30 min. The MDA content was then determined at 532 and 600 nm and expressed as nmol MDA/g FW. The content of H_2_O_2_ was determined according to Alexieva et al. (2001) with minor modifications. 0.3 g of fresh sample was mixed with 1.2 mL of 0.1% TCA. The mixture was centrifuged min at 12,000 g for 10 min at 4 °C. 250 µL of the supernatant was mixed with 250 µL of 0.1 M phosphate buffer pH 7 and 500 µL of KI buffer 1 M and incubated for 1 h in the dark. H_2_O_2_ content was then determined by measuring the absorbance at 390 nm and expressed as µmol H_2_O_2_/g FW. For the determination of MDA, H_2_O_2_, proline, and soluble sugars, the corresponding standard lines were prepared. All spectrophotometric measurements were performed on 96-well microplates using a BioTek^®^ UV-Visible Epoch™ spectrophotometer. The percentage of increase in each metabolite content in response to salt stress in MUCU16 and mutant plants was determined with respect to the metabolite content in plants of the same genotype growing under control conditions.

From the content values of metabolites with a known positive role against salinity, including proline, soluble sugars, and anthocyanins, a NaCl/control index was determined. The metabolic index was established individually for each line and tissue (leaf and root) at T11 (11 days after treatment) and calculated from the following expression:


$$\begin{array}{l} {\text{Metabolic index}}_{leaf/root}=(\frac{\text{Proline content under NaCl}}{\text{Proline content under control}}+\;\frac{\text{Soluble sugars content under NaCl}}{\text{Soluble sugars content under control}}+\\ \;\frac{\text{Anthocyanins content under NaCl}}{\text{Anthocyanins content under control}})/3 \end{array}$$

Using the same plant material, macro elements were measured in 10 g of fresh leaves and roots for the TS-1378 and MUCU16 genotypes. The measurements were performed according to the standard protocols dictated by the International Organization for Standardization (ISO) (https://www.iso.org/home.html). The macronutrients studied (phosphorus, potassium, calcium, magnesium, and sodium) were assessed by ICP-OES spectroscopy (ISO-11885). The relative content of macroelements was determined under saline conditions in relation to the content of the corresponding element under normal conditions for each genotype and tissue (leaves and roots).

### Evaluation of the potential use of TS-1378 and TS-2075 as rootstocks

A grafting experiment was conducted to evaluate the potential of the two most salt-tolerant mutant lines as rootstocks. The genetic background of the mutant collection MUCU16 was used as scion, while the mutant lines TS-1378 and TS-2075, a commercial rootstock interspecific hybrid (Camelforce, *C. maxima* x *C. moschata*) and MUCU16 itself were used as rootstocks. MUCU16/MUCU16 and ungrafted MUCU16 plants were used as controls. When the plants reached the 2–3 true leaf stage, MUCU16 scions were grafted onto the rootstocks using the cleft grafting technique. The grafted and non-grafted plants were allowed to heal and acclimate in a chamber at 24°C, 60% RH and a photoperiod of 16 h light/8 h dark, while watered with control solution until they had six true leaves. At this time (T0), the saline treatment (100 mM NaCl) was started. Various shoot growth parameters, including plant height, fresh and dry shoot weight, number of leaves, and leaf area were analyzed over time under both control and saline conditions. Three independent replicates of three to four plants each were analyzed for each genotype and irrigation condition at three different time points, 5, 7 and 11 days after treatment (T5, T7, and T11).

### Statistical analysis

Data were analyzed for multiple comparisons by analysis of variance (ANOVA) using the Statgraphic Centurion XVIII statistical software. Differences between genotypes and treatments were separated by the least significant difference (LSD) at a significance level of p ≤ 0.05.

## Results

### Identification of salinity-tolerant mutant lines by direct genetic approach

**Figure 1:**
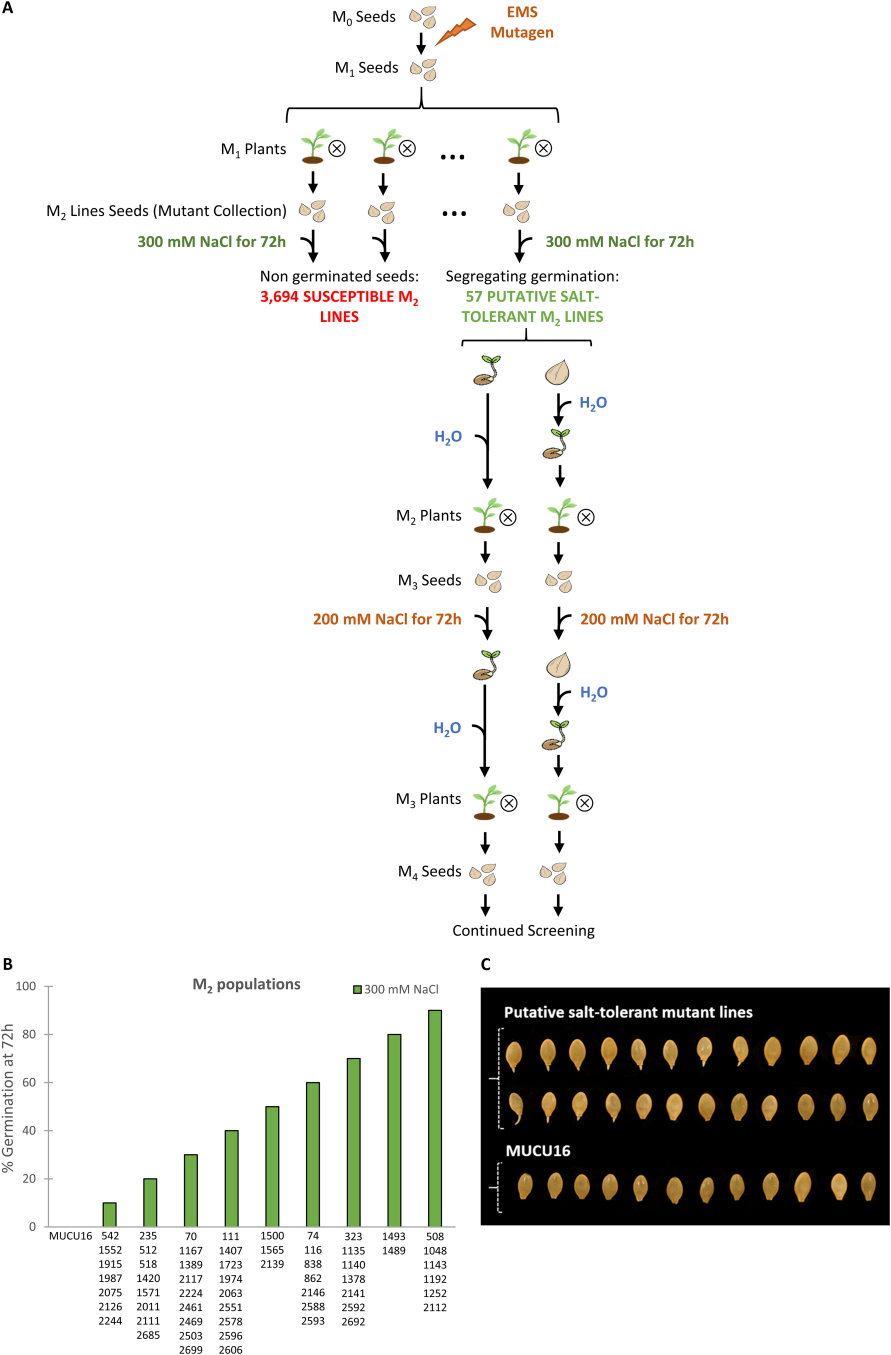
Screening of a *Cucurbita pepo* EMS collection for salt tolerance at germination. **(A)** Schematic diagram of the screening. Seeds from M_2_ lines were imbibed for 16 h in 300 mM NaCl and then allowed to germinate between two filter papers soaked in the same solution in Petri dishes. Germination was assessed every 2 h up to 72 h in two independent experiments. Lines showing any germinated seeds were selected for subsequent screening. After 72 h, seeds capable and unable to germinate under saline conditions were transferred to water for germination, and plants were then selfed to obtain the subsequent sensitive and tolerant generations. The M_3_ and M_4_ lines were evaluated at 200 mM NaCl following the same protocol. **(B)** Percentages of seed germination at 72 h under 300 mM NaCl treatment of candidate salinity-tolerant M_2_ lines. The percentage of germination was assessed in two independent experiments. Data represent means of 60 seeds per genotype. **(C)** Two examples of putative salinity-tolerant M_2_ lines identified in the screening and the genetic background MUCU16 under 300 mM NaCl at 72h. Note that candidate salt-tolerant M_2_ lines were segregating, while the MUCU16 seed was not germinated at all.

An EMS squash (*C. pepo*) mutant collection of 3,751 lines was subjected to a massive screening at the germination stage to identify salt-tolerant lines. To ensure the selection of truly resistant M_2_ lines, the screening was performed at a very high salt concentration, 300 mM NaCl, a concentration at which the genetic background of the collection MUCU16 was unable to germinate (**Supplementary Figure S1A**). The subsequent M_3_ and M_4_ generations were evaluated to ensure the inheritance of the trait using 200 mM NaCl. **Figure 1A** shows the screening across generations M_2_ through M_4_. Given that M_2_ is a segregating generation, only lines with non-tolerant and tolerant individuals were considered candidates. The M_3_ lines were obtained by self-pollination of M_2_ individuals and were phenotyped as tolerant, non-tolerant or segregated depending on the genotype of the parental M_2_ plant. The same procedure was used for M_4_ production. Only the six selected lines showed that tolerance was inherited.

Of the 3,751 lines that make up the collection, most were unable to germinate at high salt concentration and were considered susceptible lines (**Figure 1A**). Only 57 M_2_ lines were able to germinate under 300 mM NaCl in two independent experiments (tolerant lines) (**Figure 1B**). These lines showed different germination patterns after 72 h of evaluation. 24 M_2_ lines showed 50% or more germinated seeds, while 23 lines showed a lower percentage of germination. All lines with at least one germinated seed were considered candidates for tolerance at this stage of the screening (**Figure 1C**). 57 M_2_ lines showing salt tolerant and sensitive seeds were selected to generate the M_3_ and M_4_ generations.

**Figure 2:**
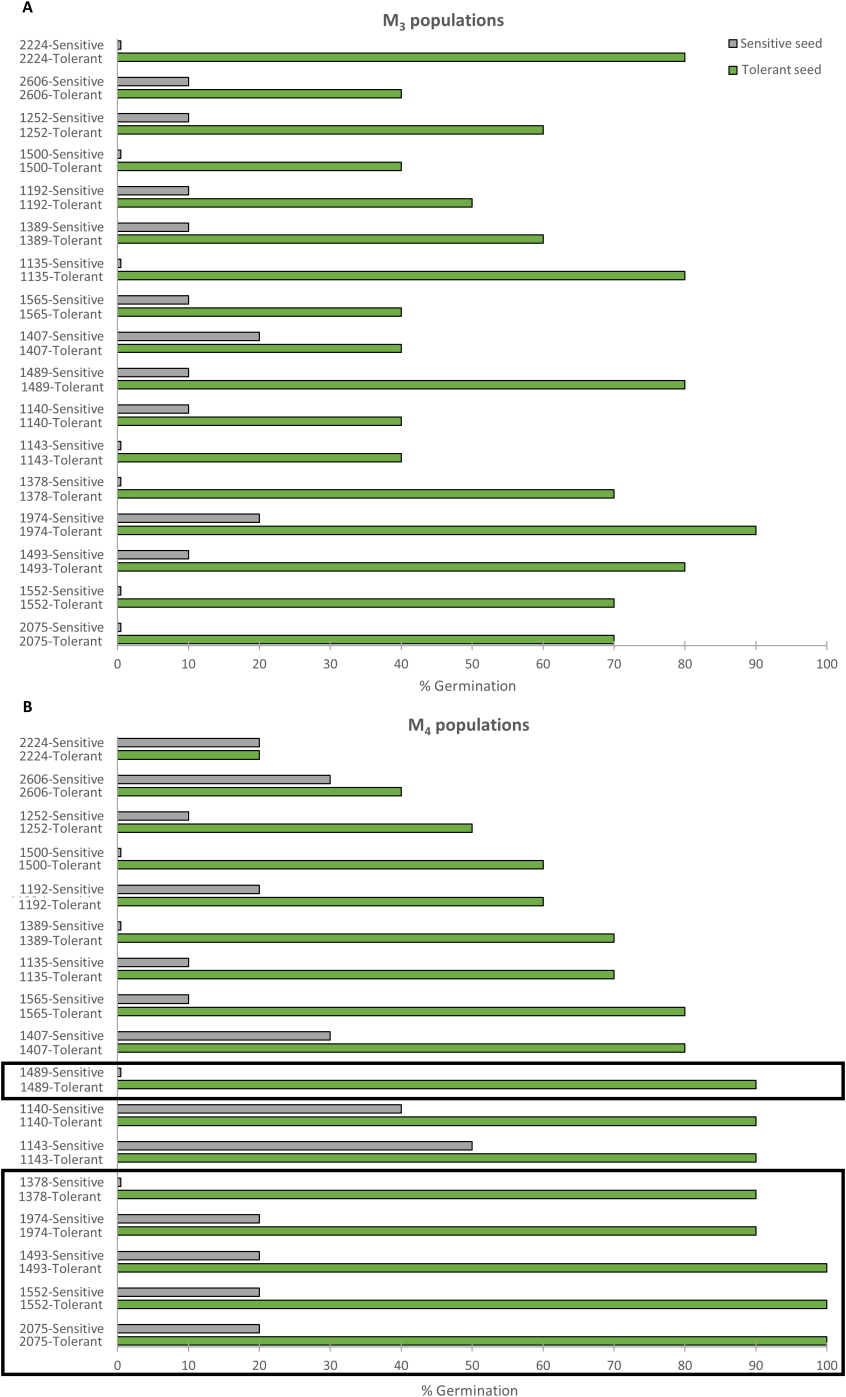
Inheritance of salinity-tolerant phenotypes. **(A)** Percentage of seed germination in sensitive (gray) and tolerant (green) M_3_ lines at 72 h under 200 mM NaCl. Sensitive and tolerant M_3_ lines were selfing progenies of M_2_ seeds that were incapable and capable of germinating under 300 mM NaCl. **(B)** Percentage of seed germination of sensitive (gray) and tolerant (green) M_4_ lines at 72 h under 200 mM NaCl. Sensitive and tolerant M_4_ lines were selfing progenies of M_3_ sensitive and tolerant lines, respectively. Data represent the means of 60 seeds per genotype. The rectangles point out the six mutant lines that were genetically stable throughout the three generations.

In the M_3_ generation, 17 lines showed higher germination when derived from a salt-tolerant M_2_ parent than when derived from a salt-sensitive M_2_ parent (**Figure 2A**). The same was true for 12 lines in the M_4_ generation (**Figure 2B**). The six most promising lines were finally selected on the basis of their maintenance of the inherited response to salt stress in the M_3_ and M_4_ generations. Under 200 mM NaCl, the selected M_3_ and M_4_ lines showed 90-100% germination when derived from selfing a salt-tolerant parent, and 0-20% germination when derived from selfing a salt-sensitive parent (**Figure 2B**). They were designated by their respective collection numbers and the prefix TS- (salt-tolerant), TS-1378, TS-1489, TS-1493, TS-1552, TS-1974, and TS-2075, and were selected for evaluation at the seedling and adult plant stages.

**Figure 3:**
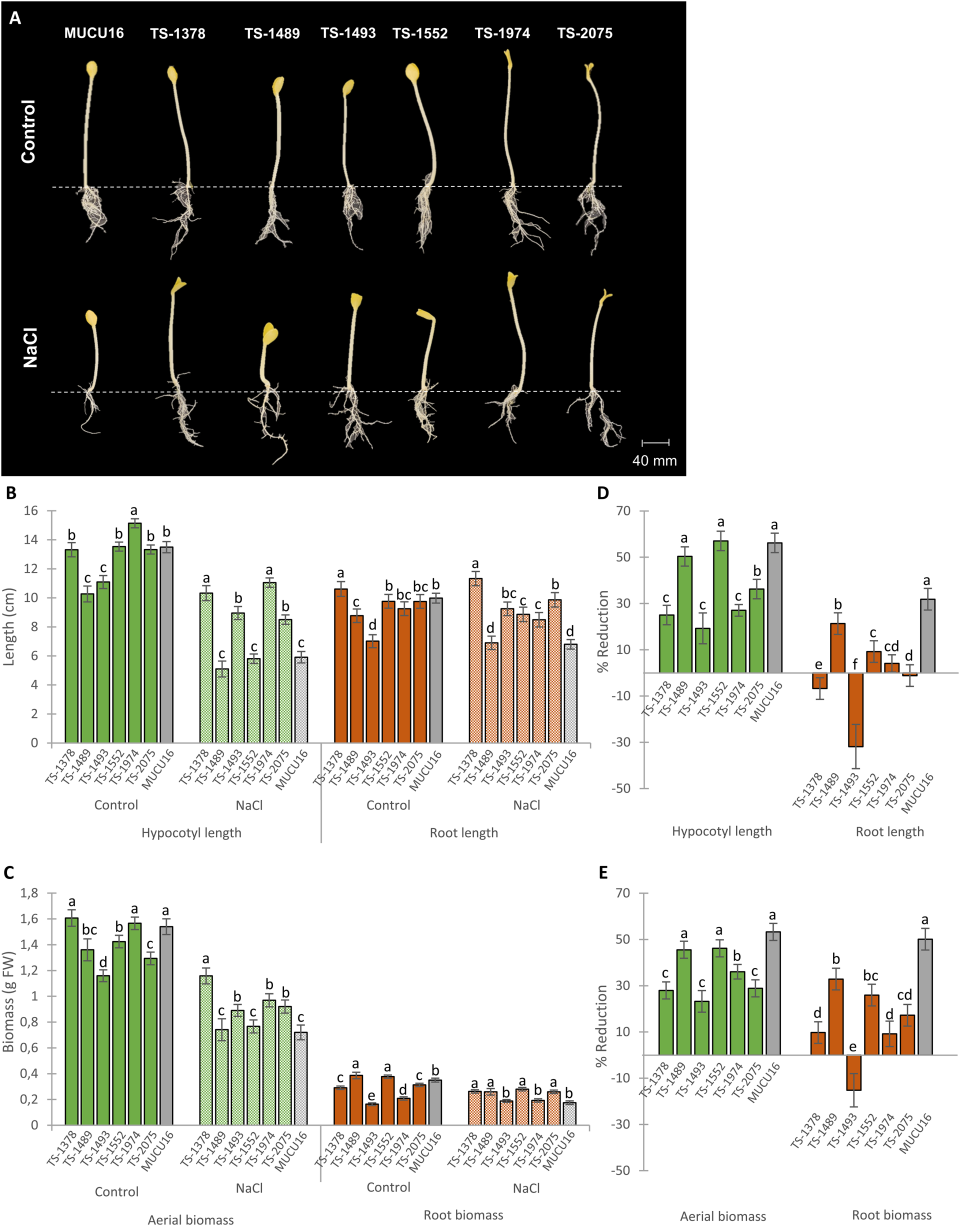
Effect of salt stress on growth parameters of MUCU16 and M_4_ mutant lines seedlings growing in darkness for 72h. **(A)** Seedlings of MUCU16 and mutant lines grown under control and 100 mM NaCl treatments for 72h. **(B)** Effect of salt stress on the length of the root and hypocotyl. **(D)** Effect of salt stress on root and aerial biomass. **(C, E)** Percentages of reduction of each growth parameter in response to salt stress in MUCU16 and mutant lines with respect to plants of the same genotype growing under control conditions. Results per genotype and treatment come from the evaluation of 15–20 seedlings. The error bars represent SE. Different letters indicate statistically significant differences (p< 0.05) between lines for the same parameter and treatment.

To investigate whether the salt-tolerant phenotype of the six mutant lines was also expressed at different stages of development, we evaluated the salt tolerance of the selected M_4_ lines at the seedling stage. **Figure 3** shows the etiolated seedlings in darkness evaluated 72 hours after planting in pots under both control and saline conditions. A dose-response study relationship between NaCl stress and seedling growth in MUCU16 was previously performed to select the optimal concentration for this assay (**Supplementary Figures S1B, C**). Among the saline concentrations tested (30, 60, 100 and 150 mM), 100 mM was chosen because it allowed a clear distinction between susceptible and tolerant phenotypes during seedling establishment.

Differences in growth parameters were observed between lines under control conditions, including plant height, aerial biomass, and root length and biomass (**Figures 3A-C**). These results indicated that the mutations altered organ growth not only under salt stress conditions, but also under normal conditions, suggesting distinct growth mechanisms that could influence the response to salt stress.

Salt treatment reduced the growth of the WT MUCU16 and all mutant lines (**Figures 3A-C**), but the mutants showed a lesser reduction in growth parameters compared to MUCU16 (**Figures 3D, E**). The salt tolerance of each mutant line was assessed by the percentage of reduction of each growth parameter in response to salt. MUCU16 showed the higher percentages of reduction, except for hypocotyl length and aerial biomass, where TS-1489 and TS-1552 were statistically equal to MUCU16 (**Figures 3D, E**). In fact, TS-1489, TS-1552, and MUCU16 reduced their hypocotyl length by more than 50%, while the rest of the mutant lines reduced it by only 19-36% (**Figure 3D**). Similarly, MUCU16, TS-1489 and TS-1552 reduced aerial biomass by more than 45%, while the rest of the mutant lines reduced it by 23-36% (**Figure 3E**). It was also found that the mutations favored a better root growth in response to salt. MUCU16 reduced root length by 31% in response to salt, while the mutant lines TS-1489, TS-1552, and TS-1974 reduced it by 21%, 9%, and 4%, respectively, and no reduction was observed in TS-2075. Notably, root length was stimulated by salt in TS-1378 (7%) and TS-1493 (31%) (**Figure 3D**). Similarly, salt treatment reduced the root biomass of MUCU16 by 50%, while the reduction in mutant lines was lower (9-32%). It is also important to note that salt stimulated the production of root biomass in line TS-1493 (15% increase compared to control conditions) (**Figure 3E**). Thus, our analysis of growth at the seedling stage indicated that the root system of all mutant lines was less sensitive to salt stress than that of the WT background. The mutant lines TS-1378 and TS-1493 stood out because their root growth was stimulated under saline conditions in terms of length (both lines) and biomass (only TS-1493).

### Effect of salt stress on WT and mutant growth parameters

**Figure 4:**
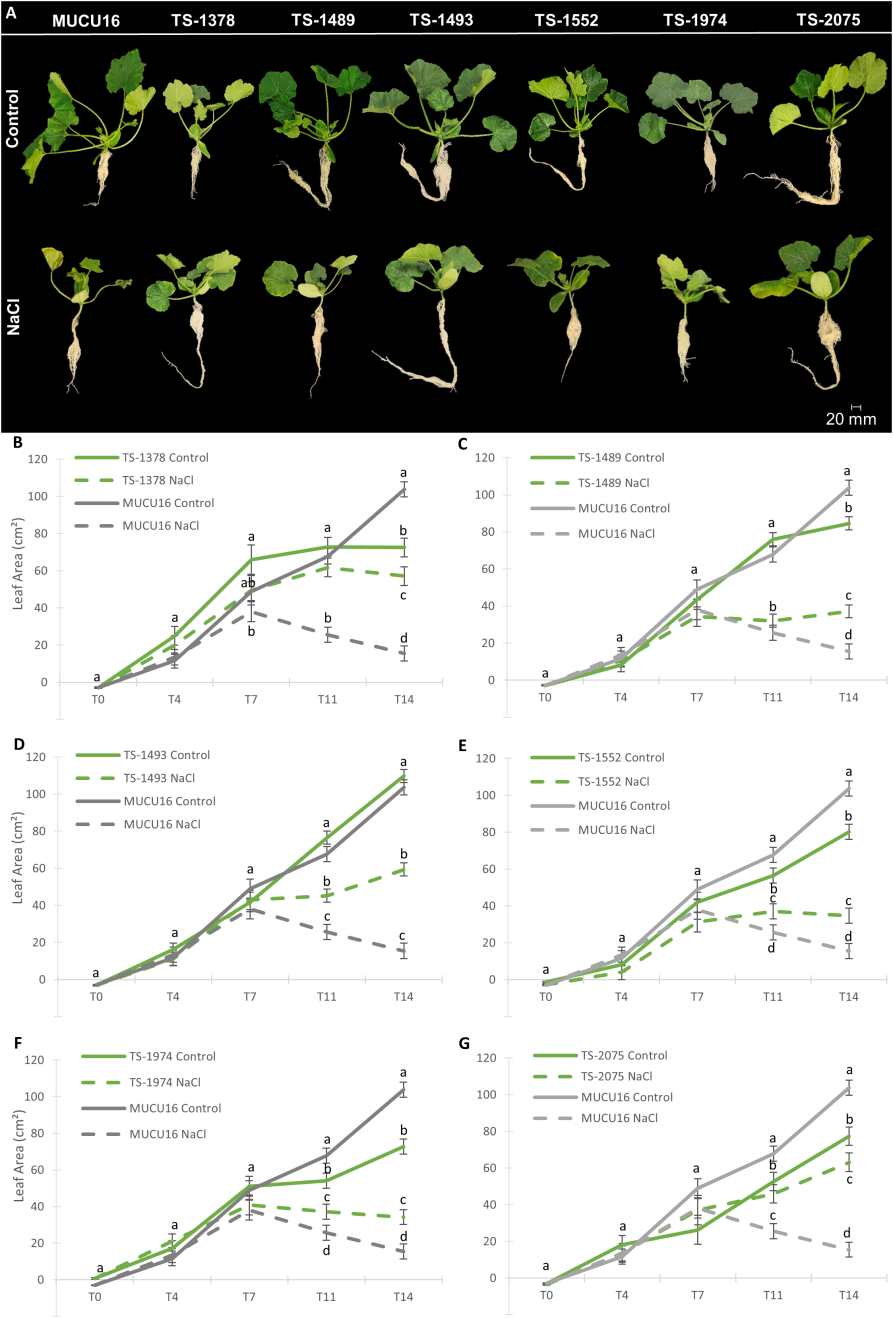
Effect of salt stress on the growth of MUCU16 and salt-tolerant M_4_ mutant plants over time. **(A)** Comparison of MUCU16 and mutant plants under control and 100 mM NaCl for 14 days. **(B-G)** Comparison of the effect of salt stress on leaf area (cm^2^) of MUCU16 and each of the TS-mutant lines over time: T0, and T4, T7, T11 and T14 (4, 7, 11 and 14 days after the beginning of the treatments). The results in each time point, genotype, and treatment come from the evaluation of 9–10 plants. Error bars represent SE. Different letters indicate statistically significant differences (p< 0.05) between samples for the same time period.

To validate the salt-tolerant phenotypes of the mutant lines, we determined the effect of salt stress on plant growth parameters at the stage of 6–7 leaf stage, including plant height, root length, vegetative and root biomass, and leaf area, at five different time points (T0, T4, T7, T11, and T14). **Figure 4** shows plants of MUCU16 and mutant lines under both control and saline treatments at T14 (**Figure 4A**), as well as the comparison of leaf area development over time between each of the mutant lines and the WT MUCU16 (**Figures 4B-G**). The remaining growth parameters are shown in **Supplementary Figures S2**-**S5**. **Supplementary Figure S6** shows the percentage of reduction of each growth parameter in response to salinity.

Mutant lines and MUCU16 showed different vigor under control conditions. At early stages (from T0 to T7), we found no significant differences in the leaf area of the mutant lines compared to the MUCU16. However, at late time points (from T11 to T14) the leaves of MUCU16 grew significantly more than those of the mutant lines under normal conditions, except for TS-1493 plants, which showed the same leaf size as the WT (**Figures 4A-G**). The same was true for aerial biomass and plant height (**Supplementary Figures S2**, **S3**). Consistent with what was observed at the seedling stage, the mutant lines did not show any growth advantage under control conditions. However, mutant lines were found to be more tolerant to salinity than the MUCU16 genetic background. At early stages, mutant lines and WT responded similarly to NaCl treatment, reducing leaf area, plant height, and aerial biomass. However, at late stages, MUCU16 showed a significantly lower leaf area and, consequently, a lower aerial biomass than the mutant lines (**Figure 4**, **Supplementary Figure S2**). In fact, MUCU16 reduced its leaf area by 85% compared to control conditions, while the mutant lines TS-1489, TS-1493, TS-1552, and TS-1974 reduced it by about 50%, and TS-1378 and TS-2075 by only 21% and 18%, respectively (**Supplementary Figure S6**). The longer the plants were exposed to salt, the more advantageous the mutant genotype was compared to WT.

MUCU16, TS-2075 and TS-1489 showed the highest root biomasses at T14 under control conditions (**Supplementary Figure S4**). Although the WT root did not show the greatest reduction in length (by 37%) in response to salt, it did show the greatest reduction in biomass (by 80%) (**Supplementary Figure S6**). Of particular interest were TS-2075 and TS-1378, which exhibited the highest root biomasses under salt stress in correlation with the lower reduction under salt stress (approximately 7 and 5 g, respectively) (**Supplementary Figures S5**, **S6**). Furthermore, these lines suffered a lower loss of plant height and leaf area in response to salt (**Supplementary Figure S6**). It was also very interesting that salt stimulated the length of the root (as in the seedling stage) and the biomass of TS-1378 (**Supplementary Figures S4**, **S5**). Taken together, these results indicated that the mutant lines were more salt-tolerant than the MUCU16 genetic background not only during germination and seedling establishment, but also at the plant stage.

### Effect of salinity on the accumulation of stress-related metabolites in WT and mutant lines

**Figure 5:**
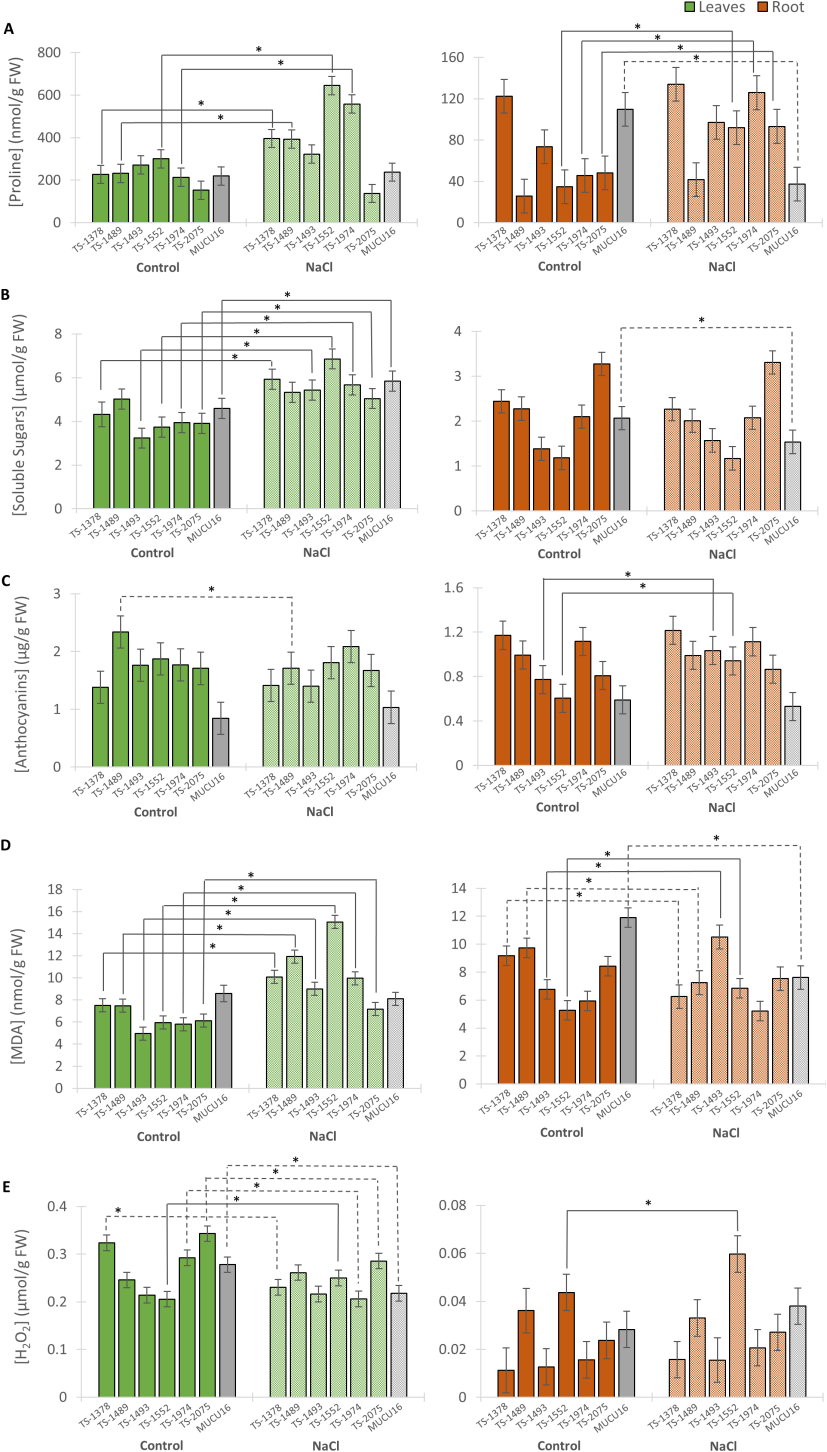
Content of stress metabolites in MUCU16 and TS-mutant plants grown under control and NaCl conditions for 11 days. **(A)** Proline, **(B)** soluble sugars, **(C)** anthocyanins, **(D)** malondialdehyde (MDA), and **(E)** hydrogen peroxide. The mean concentration of each metabolite was derived from three biological replicates of 3–4 plants each. Values are referred to organ and treatment: leaves control (dark green), leaves NaCl (light green), roots control (dark brown) and roots NaCl (light brown). For each parameter, the WT MUCU16 was shown in gray. Error bars represent SE. The asterisk indicates statistically significant differences (p< 0.05) between the control and salt-treated samples of the same genotype. Significant increases or decreases in metabolite content in response to saline treatment are indicated with solid or dashed lines, respectively.

To investigate the mechanisms that could be responsible for the differential growth of WT and mutant plants under salinity, the levels of several stress-related metabolites were measured in plants at T11 (11 days after the start of treatment). This time point was chosen based on the development of plant growth over time. Since at T14 the growth of MUCU16 was totally affected, T11 was chosen because the plant was not yet in the critical phase, but the biochemical response was expected to be already active. **Figure 5** shows the content of proline and soluble sugars, widely known for their osmoprotective function, anthocyanins, a flavonoid with antioxidant capacity, as well as MDA and H_2_O_2_, which measure membrane damage, in both leaves and roots of the seven lines. **Supplementary Figure S6** shows the percentage of increase of each metabolite in response to salinity.

In leaves, the mutant lines and the WT genotype showed the same accumulation of proline and soluble sugars under control conditions. However, under saline conditions, five of the six mutant lines showed a significantly higher proline content than MUCU16, with an increasing ratio between 19 and 161% (**Figure 5A**, **Supplementary Figure S6**). Of particular interest were the lines TS-1552 and TS-1974, which showed the highest proline content under saline conditions with values of 644 and 558 nmol/g of FW, respectively (**Figure 5A**). In response to salt treatment, most mutant lines increased their leaf soluble sugar content between 38 and 83%, while MUCU16 increased it by 27% and the line TS-1489 did not change (**Figure 5B**, **Supplementary Figure S6**). Under control conditions, the mutant lines showed the highest anthocyanin content compared to that of the WT. However, the anthocyanin content did not change in response to salt for any of the lines, except for TS-1489, which significantly decreased the anthocyanin content in response to salt (**Figure 5C**). Under control conditions, the leaf MDA content was significantly lower in all mutant lines than in the WT, except for lines TS-1378 and TS-1489. In response to salt, the six mutant lines significantly increased leaf MDA content, while the WT did not (**Figure 5D**). H_2_O_2_ varied among the lines under control conditions, being higher in TS-1378 and TS-2075 than in MUCU16. Notably, it was reduced or maintained in most lines (except TS-1552) in response to salt treatment (**Figure 5E**, **Supplementary Figure S6**).

Since the root remains in primary contact with salt, we also analyzed the metabolite content in this organ and observed a distinct metabolic behavior between the lines under both control and saline conditions. All lines except WT MUCU16 increased proline content from 32 to 175% (TS-1552, TS-1974 and TS-2075) or kept it constant (TS-1378, TS-1489 and TS-1493) after 11 days of salt exposure (**Figure 5A**, **Supplementary Figure S6**). In contrast to what was observed in leaves, soluble sugars in roots remained the same in all mutant lines, while in MUCU16 soluble sugars decreased by 25% (**Figure 5B**, **Supplementary Figure S6**). Similarly, there were differences in anthocyanin content between lines growing under control conditions. After salt treatment, lines TS-1493 (34% increase) and TS-1552 (56% increase) responded by increasing the content of this metabolite, while the rest of the mutant lines and MUCU16 kept it constant (**Figure 5C**, **Supplementary Figure S6**). Under control conditions there were differences in the levels of MDA and H_2_O_2_ between the roots of the seven lines, with the MUCU16 root having the highest level of MDA. Salt treatment caused an increase in MDA in roots of TS-1493 and TS-1552, which had the lowest levels in the control (**Figure 5D**, **Supplementary Figure S6**). The mutant lines TS-1378 and TS-1489, together with MUCU16, decreased the MDA content in response to salt, while TS-1974 and TS-2075 kept it constant. Interestingly, line TS-1552 responded to salt by increasing the level of H_2_O_2_ (**Figure 5E**).

In summary, the salinity tolerance phenotype of the mutant lines was generally associated with the levels of proline, soluble sugars, and MDA. MDA increased in the leaves of all lines, proline increased or maintained levels compared to control conditions, soluble sugars maintained levels compared to control conditions, and anthocyanins increased their levels in the root of two mutant lines.

**Figure 6:**
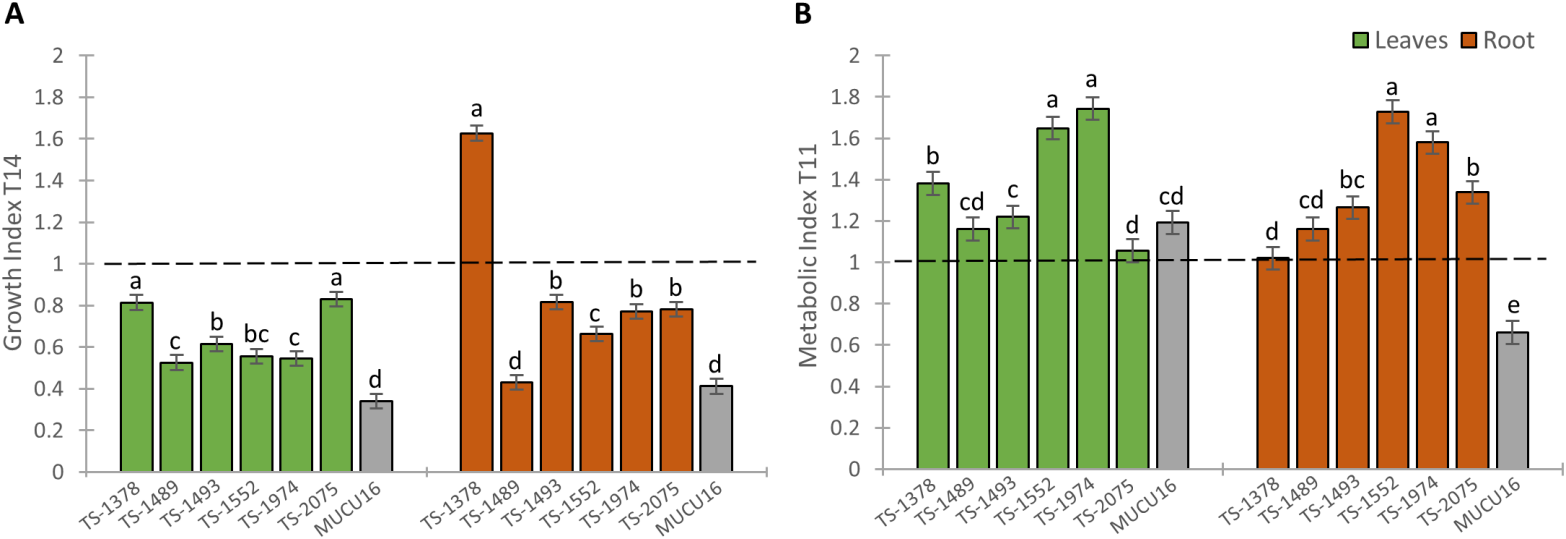
Comparison of the effect of NaCl stress on plant growth and metabolite production response between MUCU16 and TS-mutant lines. **(A)** Growth index (GI) integrating growth parameters at T14. **(B)** Metabolic index (MI) integrating the content of metabolites at T11. Leaves (dark green) and roots (dark brown). Error bars represent SE. Different letters indicate statistically significant differences (p< 0.05) between lines for the same tissue.

We generated two indices that integrated either growth or biochemical parameters in WT and mutant plants (**Figure 6**). The growth index (GI) indicated whether plant growth decreased or increased in response to salinity and allowed the classification of mutant lines based on their sensitivity or tolerance to salt in terms of growth. Although all mutant lines showed sensitivity to salt stress (GI<1), the GI in shoots of the TS-1378 and TS-2075 mutant lines was close to 1 (0.81 and 0.83, respectively), while the MUCU16 shoot showed the highest sensitivity (GI = 0.34). The same was true for root growth, with MUCU16 (GI = 0.41) and TS-1489 (GI = 0.43) being the most sensitive genotypes to salinity. In contrast, roots of TS-1378, TS-1493, TS-1974 and TS-2075 showed the lowest sensitivity to salt stress, with growth indices ranging from 0.77 to 1.62. Line TS-1378 showed the better response, with a root GI value of 1.62 (**Figure 6A**).

On the other hand, the metabolic index indicated whether the content of metabolites increased or decreased in response to salt treatment. The shoot metabolic index (SMI) was found to be >1 in all seven lines, indicating that the content of osmoprotective and antioxidant metabolites in the leaves of all lines increased in response to salt. The highest values were observed in TS-1974 (1.74), TS-1552 (1.65) and TS-1378 (1.38), while the lowest values were observed in TS-1493 (1.22), TS-1489 (1.16) and TS-2075 (1.05) as well as in the salt-sensitive MUCU16 (1.19). All mutant lines showed a root MI>1 (1.02-1.72), with maximum values in TS-1552 (1.72) and TS-1974 (1.58). In contrast, MUCU16 showed a root MI of 0.66 (**Figure 6B**). It should be noted that the sensitive genotype MUCU16 induced the production of osmoprotectants in leaves, but not in roots, in response to salinity, whereas the mutant lines induced their production in both tissues. Lines TS-1378 and TS-2075 were selected for further analysis because they were the best lines in terms of growth parameters, in addition to showing valuable induction of metabolites in shoot and root.

Since salt stress was found to stimulate root growth and improve the aerial response of TS-1378 plants, we compared the ion content of this salt-tolerant genotype with that of the salt-sensitive WT genotype MUCU16. **Table 1** shows the effect of salinity on the nutrient content of leaves and roots. Under control conditions, the greatest differences were found in roots, organ in which TS-1378 had higher levels of K^+^, Ca^2+^, Mg^2+^ and Na^+^, and lower levels of P^3-^. No differences were observed in leaves, except for P^3-^, which was reduced, and Ca^2+^, which was increased in the mutant plants. Salt caused an imbalance in ion content in both lines, decreasing Ca^2+^ and Mg^2+^ in roots of TS-1378 and increasing K^+^ in MUCU16. In leaves, Ca^2+^ and Mg^2+^ were increased in MUCU16, and K^+^ in TS-1378. The phytotoxic ion Na^+^ was higher in the roots of TS-1378 compared to those of MUCU16 under control conditions, but was the same in the leaves. However, salt stress caused an increase in Na^+^ content in roots that was 2.30 times higher in MUCU16, but only 1.65 times higher in TS-1378. The K^+^/Na^+^ ratio was observed to be 27.99 in MUCU16 leaves and 21.94 in those of TS-1378, but salt treatment reduced them to equal values (≈3.20). On the contrary, the K^+^/Na^+^ ratio in roots grown under control conditions indicated an increased content of K^+^ with respect to Na^+^ in TS-1378 (1.32) compared to those of the WT (1.02). Furthermore, although salt treatment decreased the K^+^/Na^+^ ratio in the roots of both genotypes, it remained higher in TS-1378, suggesting an improved response to salt of this mutant.

**Table 1:** Content of macronutrients and phytotoxic elements in MUCU16 and TS-1378 mutant leaves and roots of plants grown under control and NaCl conditions 11 days after treatment.

	Macronutrients (mg/kg)				Phytotoxic elements (mg/kg)	
	P^3-^	K^+^	Ca^2+^	Mg^2+^	Na^+^	K^+^/Na^+^ Ratio
Leaves						
MUCU16 Control	824,5 a	3541 b	417,5 c	775,5 c	126,5 b	27,99 a
MUCU16 NaCl	796 a (0,97)	3423,5 b (0,97)	677,5 b (1,62)	1622,5 a (2,09)	1047 a (8,28)	3,27 c
TS-1378 Control	613,5 b	3433,5 b	719,5 ab	1043 bc	156,5 b	21,94 b
TS-1378 NaCl	664,5 b (1,08)	4137 a (1,20)	995,5 a (1,38)	1406,5 ab (1,35)	1294,5 a (8,27)	3,20 c
Root						
MUCU16 Control	570 a	1542 c	211 b	724 b	1512 c	1,02 b
MUCU16 NaCl	587 a (1,03)	2409 b (1,56)	216,5 b (1,03)	628 b (0,87)	3484,5 a (2,30)	0,69 d
TS-1378 Control	480 b	3002,5 a	322,5 a	2754 a	2269 b	1,32 a
TS-1378 NaCl	519,5 ab (1,08)	2748,5 ab (0,92)	224,5 b (0,70)	877,5 b (0,32)	3747,5 a (1,65)	0,73 c

The relative content of elements under saline conditions in relation to the content of the corresponding element under control conditions is expressed in parenthesis for each genotype and tissue. Different letters within the same column and tissue indicate significant differences between means (p< 0.05).

### Evaluation of TS-1378 and TS-2075 tolerant mutant lines as squash rootstocks

**Figure 7:**
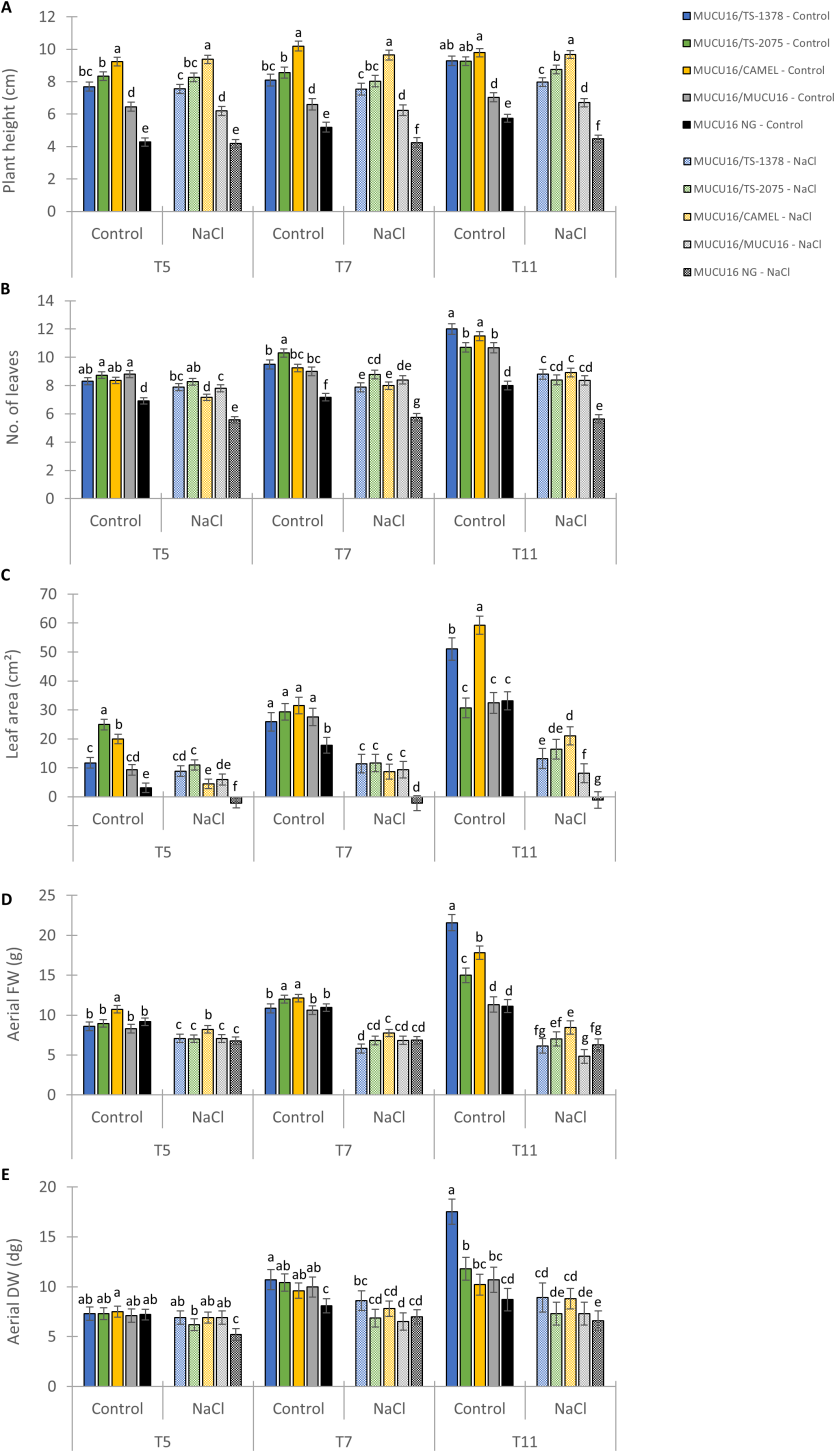
Effect of salt stress on aerial growth parameters of MUCU16 scions grafted into MUCU16 (salt-sensitive), and mutant lines TS-1378 and TS-2075 (salt-tolerant). The non-grafted MUCU16 plants and those grafted into the commercial rootstock Camelforce (CAMEL, interspecific hybrid *C. moschata* x *C. maxima*) were used as control. **(A)** Plant height (cm), **(B)** Number of leaves, **(C)** Leaf area (cm²), **(D)** Aerial fresh weight (FW, g) and **(E)** Aerial dry weight (DW, dg). The parameters were evaluated in at least three biological replicates per genotype and treatment, each composed of a set of 3–4 different plants and three technical replicates, at three different time points, 5 (T5), 7 (T7) and 11 (T11) days after the beginning of treatment (100 mM NaCl). NG, non-grafted. Error bars represent SE. Different letters indicate statistically significant differences (p< 0.05) between lines.

Grafting onto suitable rootstocks is an important technique for the appropriate cultivation of some Cucurbitaceae species (Schwarz et al., 2010). Based on growth and metabolic indices (**Figure 6**), the most tolerant lines were TS-1378 and TS-2075 mutants. Therefore, they were selected to evaluate their potential use as rootstocks for salinity tolerance. MUCU16 was grafted onto WT MUCU16 and the mutant lines, and growth parameters were evaluated at 5, 7 and 11 days after the start of salinity treatment (100 mM). Non-grafted MUCU16 and MUCU16 grafted into the commercial rootstock Camelforce (CAMEL) were used as negative and positive controls, respectively. **Figure 7** shows the effect of grafting, as well as the effect of the different genotypes on the aerial growth of MUCU16 scions under both normal and saline conditions. The difference for each parameter was accentuated over time due to salinity, but there was a significant effect of grafting and genotypes. Therefore, grafting improved plant height, number of leaves, and leaf area regardless of genotype compared to non-grafted plants under normal and saline conditions (**Figures 7A-C**). In fact, the differences observed 11 days after saline treatment indicated that only non-grafted MUCU16 and MUCU16/TS-1378 reduced their plant height in response to salinity. A better response to salinity was observed in MUCU16/CAMEL, followed by MUCU16/TS-2075 and MUCU16/TS-1378 (**Figure 7A**).

On the other hand, salinity reduced the number of leaves in all grafted and non-grafted plants, and there were no differences between genotypes. (**Figure 7B**). The greatest differences were observed in relation to the effect of the genotype on leaf area. Under control conditions, MUCU16/CAMEL and MUCU16/TS-1378 stood out as having the largest leaf area after 11 days. However, the response under salinity conditions was similar for the positive control (CAMEL) and experimental rootstocks (TS-2075 and TS-1378), with a significant improvement in leaf area compared to MUCU16/MUCU16 and ungrafted plants, but with no significant differences between them (**Figure 7C**). Interestingly, at the end of the experiment, MUCU16/TS-1378 had the highest fresh and dry weight under control conditions, indicating that the plants had greater biomass in addition to greater water uptake. However, under saline conditions, the differences between MUCU16/TS-1378 and non-grafted and MUCU16/MUCU16 plants were attenuated (**Figures 7D, E**).

## Discussion

### High-throughput screening at the germination stage is effective for identifying salt-tolerant EMS mutants

Salinity affects seed germination by impairing water uptake and increasing the presence of toxic elements that alter metabolism, hormonal signaling, and cell division and elongation (Parihar et al., 2015). Therefore, germination is the first process of plant development involved in the response to salt stress (Van Zelm et al., 2020). Taking advantage of this, we designed a high-throughput screen at the germination stage to detect salt-tolerant EMS mutants in *C. pepo*. EMS is a widely used mutagenic agent that induces random point mutations in DNA than can produce phenotypes of agronomic interest. To discover these phenotypes, mutant collections should be screened using an appropriate phenotyping method. In this regard, screenings at the germination stage are advantageous because they allow the evaluation of a large number of mutant lines by reducing time and space. Indeed, other salt-tolerant EMS mutants have already been identified in Arabidopsis (Quesada et al., 2000) and wheat (Lethin et al., 2020) using a similar strategy. Seeds with extremely contrasting phenotypes (able and unable to germinate at 300 mM NaCl) of candidate M_2_ lines were selfed to obtain tolerant and sensitive M_3_ and M_4_ lines, which also allowed to establish the inheritance of the trait under study. Finally, six mutant lines were selected whose salt tolerance phenotype was maintained in M_2_, M_3_ and M_4_ generations, demonstrating that the germination-based screening method is convenient and effective for the identification of salt-tolerant mutant lines.

Because tolerance during germination may not be associated with tolerance at later developmental stages (Dewey, 1962; Shannon, 1985; Flowers, 2004), the salt-tolerant phenotype of our six cucurbit mutants was also validated during seedling and plant establishment. Previous studies also identified salt-tolerant EMS mutants using a germination-based method (Quesada et al., 2000; Lethin et al., 2020). In contrast, salt-tolerant mutants in *C. moschata* and soybean were detected at the seedling stage (Chen et al., 2018; Moon et al., 2023), and rice mutants were selected based on an evaluation in adult plants (Takagi et al., 2015). However, screening methods based on plant stages undoubtedly require more time and infrastructure and reduce the number of lines to be evaluated. Therefore, we propose here a rapid and efficient method that allows high-throughput screening of large collections of plants for salt tolerance.

### Mechanisms of salt tolerance in *C. pepo* EMS mutants

The first effect of salinity on plant development is a reduction in vegetative growth (Munns, 2002), which is observed in two phases: a rapid effect due to external osmotic pressure in the root, and a slower effect due to ion toxicity (Isayenkov and Maathuis, 2019). To cope with salt imbalance, plants have developed mechanisms to regulate osmotic homeostasis, exclude toxic ions from the shoot, and remove toxic ions from specific tissues, cells, and subcellular organelles (Munns and Tester, 2008). In this paper, we present six EMS mutant lines that have been shown to have improved growth and increased osmolyte production under salt stress compared to the sensitive WT genotype.

Among the diversity of plant strategies in the face of salt stress, a relevant result was the smaller plant size of the *C. pepo* EMS mutants compared to MUCU16 when grown for 11–14 days under control conditions. MUCU16 showed higher plant height, root and leaf biomass, and leaf area than most of the EMS mutant lines after 14 days of experiment. However, mutant plants were more tolerant to salt stress (100 mM NaCl) at T14 than MUCU16 in terms of growth. A reduction in leaf area can be considered as an avoidance mechanism by reducing the number of stomata and minimizing water loss through transpiration (Savé et al., 1994; Ruiz-Sánchez et al., 2000). In addition, this strategy could favor the retention of toxic ions in the roots, limiting their accumulation in the photosynthetic parts of the plant (Colmer et al., 2005; Munns and Tester, 2008).

Interestingly, all identified mutant lines except TS-2075 showed less root biomass than MUCU16 under standard conditions, but less reduction in root biomass in response to salt, suggesting that a smaller root system may be advantageous in coping with salinity. This salt tolerance strategy has already been reported in other species. For example, the Arabidopsis loss-of-function ethylene mutant *etr1–7* showed reduced root length under control conditions, but had longer roots than the WT at 150 mM and 200 mM NaCl, an opposite phenotype to the gain-of-function mutant *etr1-1* (Wang et al., 2008). Therefore, the positive role of ethylene in salt tolerance is associated with smaller root size under standard conditions. The drought tolerance of the brassinosteroid mutant *bri1* of *Brachypodium distachyon* is associated with a reduced root system (Feng et al., 2015). Similarly, the salt tolerance of the squash brassinosteroid-deficient mutant *dwfcp* is associated with a reduced root size compared to WT (Alonso et al., 2024). Since the root system requires more than 50% of assimilates for respiration (Liu et al., 2004), a smaller root could lead to a greater availability of resources to cope with stress (Ma et al., 2010).

Of particular interest was the mutant TS-1378, which showed increased root length and biomass in response to salt. Root and shoot biomass can be an indicator of salt tolerance when accompanied by other parameters, such as the accumulation of valuable ions (Dos Reis et al., 2012). TS-1378 showed improved root growth along with interesting results regarding ion content, especially Na^+^ and K^+^. In addition to nutrient imbalance and Na^+^ toxicity, salinity prevents K^+^ accumulation in the cell, which is required for several essential enzymatic reactions, protein synthesis and ribosome function (Sardans and Peñuelas, 2021). Similarly, salt stress induces changes in Ca^2+^ metabolism that may play an important role in salinity tolerance. In Arabidopsis and rice, Ca^2+^ regulates K^+^/Na^+^ homeostasis (Shabala et al., 2006; Wu and Wang, 2012). Significantly, we observed that the root of TS-1378 accumulated less Na^+^ than that of MUCU16 after 11 days of salt exposure compared to the control conditions. In addition, the Ca^2+^ content was higher in the mutant root under control conditions, and the K^+^/Na^+^ ratio was also higher in the mutant under both control and salt conditions compared to the WT. These results suggest that TS-1378 roots have the ability to reduce the prevention of K^+^ accumulation under salt stress.

In general, the salt tolerance of the mutant lines was correlated with improved production of metabolites such as proline, soluble sugars, and anthocyanins. These osmolytes are known to have a protective function by restoring osmotic homeostasis, cell volume and turgor, and by scavenging ROS to protect membranes and proteins (Sharma et al., 2011). Numerous works using overexpression of osmolyte biosynthesis genes, exogenous treatments and osmolyte deficient mutants have demonstrated the positive role of proline and sugars in salt tolerance (Abebe et al., 2003; Khedr et al., 2003; Baea et al., 2005; Simon-Sarkadi et al., 2006; Conde et al., 2007; Iordachescu and Imai, 2008; Nounjan et al., 2012). On the other hand, flavonoids such as anthocyanins are also important as antioxidants in the response of plants to salinity (Ahmad et al., 2009). Overexpression of the anthocyanin pigment gene *PAP1* shows increased salt tolerance (Oh et al., 2011). Cebrián et al. (2021) also showed that the enhanced salt tolerance of the ethylene-insensitive pumpkin mutant *etr2b* correlates with increased leaf production of proline, sugars, and anthocyanins.

We have also found that while the production of proline and soluble sugars was enhanced during the salt response in both leaves and roots, MDA was found to be specific for the leaves and anthocyanins for the roots. MDA content is commonly associated with the degree of membrane damage under biotic and abiotic stresses (Sudhakar et al., 2001; Neto et al., 2006) and has been found to be induced by salinity in many species (Singh et al., 2021; Yasir et al., 2021; Wang et al., 2022). Sensitive cultivars accumulated more MDA in eggplant seedlings (Hannachi and Van Labeke, 2018), and reduced MDA accumulation was associated with improved growth under salinity stress in tomato plants (Ahanger et al., 2019). In contrast, Morales and Munné-Bosch (2019) proposed that a decrease in MDA levels during stress indicates an adequate regulation of redox signaling and, thus a process of acclimation rather than damage. From this perspective, MDA would play a beneficial role by activating regulatory genes involved in plant defense and development. In support of this idea, foliar MDA levels were transiently induced in salt-stressed rosemary plants, along with the activation of the antioxidant system that scavenges ROS (Tounekti et al., 2011). Consistent with this, we found a higher salt-induced MDA levels in mutant leaves than in WT leaves, but fewer symptoms of stress damage.

### TS-1378 and TS-2075 salt tolerance mutants have rootstock potential

Grafting is an appropriate technique to address soil challenges such as salinity. The use of salt-tolerant rootstocks to improve crop growth and yield under saline conditions has been previously reported in Clemenules mandarin trees (Navarro et al., 2010) and pepper (Penella et al., 2015; 2016). In cucurbits, interspecific hybrids of *Cucurbita* (*C. maxima* x *C. moschata*) can be used as rootstocks to improve salt tolerance of melon scions (Ulas et al., 2020). In cucumber, salt-tolerant luffa or native bottle gourd rootstocks have been used (Guo et al., 2023; Abbas et al., 2024). Here, we report the use of two salt-tolerant *C. pepo* genotypes, TS-1378 and TS-2075, as rootstocks. They improve the plant scion growth to a level comparable to the commercial hybrid Camelforce (*C. maxima* x *C. moschata*). Camelforce is widely used as rootstock for cucurbits and has been shown to improve the growth and yield of melon (Toporek and Keinath, 2020) and watermelon crops (Ricárdez-Salinas et al., 2010). Of note is the positive effect of TS-1378 on fresh and dry aerial biomass, which exceeds even the commercial rootstock Camelforce.

Regardless of genotype, our experiments showed that grafting limited the effect of salt on plant growth. As Camelforce, the TS-1378 and TS-2075 mutant lines improved the salinity response of grafted MUCU16 scions in terms of plant height (T5-T11), leaf area (T11), and dry weight (TS-1378, T11). Significantly, we found that MUCU16 induced the production of osmoprotectants in leaves, but not in the roots. However, the mutant genotypes induced or maintained the production of proline, soluble sugars, and anthocyanins in both leaves and roots. Since osmolytes contribute to osmotic adjustment (Chen and Jian-Guo, 2010), the higher accumulation of osmolytes observed in the roots of the mutant lines could be beneficial for the growth of the grafted plants. In addition, the favorable K^+^/Na^+^ ratio found in the TS-1378 roots could contribute not only to osmotic adjustment, but also to plant growth under salinity due to the valuable functions of K^+^ in cell metabolism (Sardans and Peñuelas, 2021).

## Conclusions

High throughput screening in *C. pepo* identified six EMS lines with high salt tolerance at germination. The salt tolerance of these lines was also confirmed at the seedling and 6–7 true leaf plant stages. All mutant lines showed reduced plant size when grown under standard conditions, but an improved response to salt in terms of growth. In addition, the salt-tolerant mutants showed higher accumulation of protective osmolytes under salt stress and, when used as rootstocks, TS-1378 and TS-2075 improved scion growth to a level comparable to commercial *C. maxima* x *C. moschata* interspecific hybrids. In summary, this research not only provides comprehensive insights into plant responses to salt stress, but also reports novel squash mutants that could be important genetic resources for improving salt tolerance in commercial cultivars of *C. pepo*.

## Data Availability

The original contributions presented in the study are included in the article/**Supplementary Material**. Further inquiries can be directed to the corresponding authors.

## Supplementary material

Supplementary Figure 1 Dose-response relationship between NaCl concentration and germination and seedling growth in the genetic background of the collection, MUCU16. **(A)** Effect of different concentrations of NaCl on the percentage of germination of MUCU16 at 72 h. The seeds were imbibed for 16 h at 24 °C and then allowed to germinate between two filter papers soaked in the same solution. The data represent means of 60 seeds per treatment. **(B, C)** Effect of different concentrations of NaCl on root and hypocotyl length **(B)** and biomass **(C)** of seedlings grown in darkness for 72 h. The parameters were evaluated in three biological replicates per treatment, each composed of a set of 3–4 different plants. Circles indicate the concentrations selected for further experiments. The error bars represent SE. Different letters indicate statistically significant differences (p< 0.05) between the samples of each parameter evaluated.

Supplementary Figure 2 Effect of salt stress on aerial biomass of MUCU16 and salt-tolerant mutant lines over time. **(A-F)** Comparison of the effect of salt stress on aerial biomass (g) of MUCU16 and each of the TS-mutant lines over time: T0 and T4, T7, T11, and T14 (4, 7, 11 and 14 days after starting the treatments). The results for each time point, genotype, and treatment come from the evaluation of 9–10 plants. The error bars represent SE. Different letters indicate statistically significant differences (p< 0.05) between samples for the same time period.

Supplementary Figure 3 Effect of salt stress on hypocotyl length of MUCU16 and salt-tolerant mutant lines over time. **(A-F)** Comparison of the effect of salt stress on the hypocotyl length (cm) of MUCU16 and each of the TS-mutant lines over time: T0 and T4, T7, T11, and T14 (4, 7, 11 and 14 days after starting treatments). The results for each time point, genotype, and treatment come from the evaluation of 9–10 plants. The error bars represent SE. Different letters indicate statistically significant differences (p< 0.05) between samples at the same time.

Supplementary Figure 4 Effect of salt stress on root biomass of MUCU16 and salt tolerant mutant lines over time. **(A-F)** Comparison of the effect of salt stress on root biomass (g) of MUCU16 and each of the TS-mutant lines over time: T0 and T4, T7, T11, and T14 (4, 7, 11 and 14 days after the beginning of the treatments). The results for each time point, genotype, and treatment come from the evaluation of 9–10 plants. The error bars represent SE. Different letters indicate statistically significant differences (p< 0.05) between samples at the same time.

Supplementary Figure 5 Effect of salt stress on root length of MUCU16 and salt-tolerant mutant lines over time. **(A-F)** Comparison of the effect of salt stress on root length (cm) of MUCU16 and each of the TS-mutant lines over time: T0 and T4, T7, T11, and T14 (4, 7, 11 and 14 days after the beginning of the treatments). Results in each time point, genotype and treatment come from the evaluation of 9–10 plants. Error bars represent SE. Different letters indicate statistically significant differences (p< 0.05) between samples at the same time.

Supplementary Figure 6 Effect of salt stress on growth parameters and metabolites content of MUCU16 and salt-tolerant mutant plants. **(A)** Percentages of reduction at T14 (14 days after the beginning of the experiment) of each morphological parameter (root and hypocotyl length, aerial and root biomass, and leaf area) in response to salt stress. **(B, C)** Percentages of increase at T11 (11 days after the beginning of the experiment) of metabolite content (proline, soluble sugars, anthocyanins, MDA and H_2_O_2_) in response to salt stress of MUCU16 and mutant **(B)** leaves (dark green) and **(C)** root (dark brown). The genotype MUCU16 is always represented in gray. The percentages were calculated with respect to plants of the same genotype growing under control conditions. The error bars represent SE. Different letters indicate statistically significant differences (p< 0.05) between lines for the same parameter/metabolite.
