# Supplementary figures and images for: High throughput screening and evaluation of salt-tolerant mutants from an EMS collection of Cucurbita pepo
Source: Front Plant Sci. 2025 May 15;16:1548576. doi: 10.3389/fpls.2025.1548576 (PMC12119591; doi:10.3389/fpls.2025.1548576)

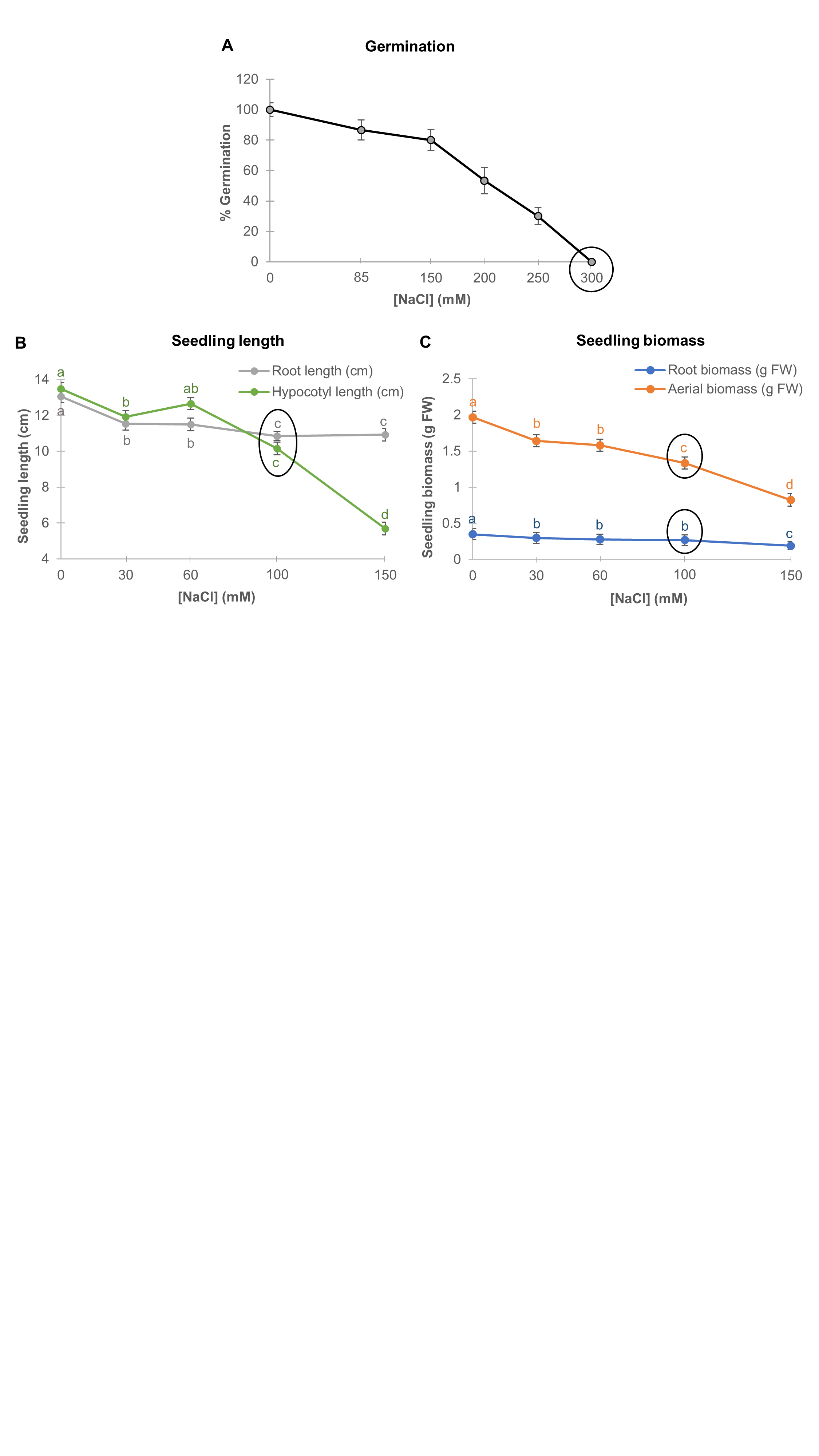

Supplement: Supplementary Figure 1 — Dose-response relationship between NaCl concentration and germination and seedling growth in the genetic background of the collection, MUCU16. (A) Effect of different concentrations of NaCl on the percentage of germination of MUCU16 at 72 h. The seeds were imbibed for 16 h at 24 °C and then allowed to germinate between two filter papers soaked in the same solution. The data represent means of 60 seeds per treatment. (B, C) Effect of different concentrations of NaCl on root and hypocotyl length (B) and biomass (C) of seedlings grown in darkness for 72 h. The parameters were evaluated in three biological replicates per treatment, each composed of a set of 3–4 different plants. Circles indicate the concentrations selected for further experiments. The error bars represent SE. Different letters indicate statistically significant differences (p< 0.05) between the samples of each parameter evaluated. [file Image1.tif]

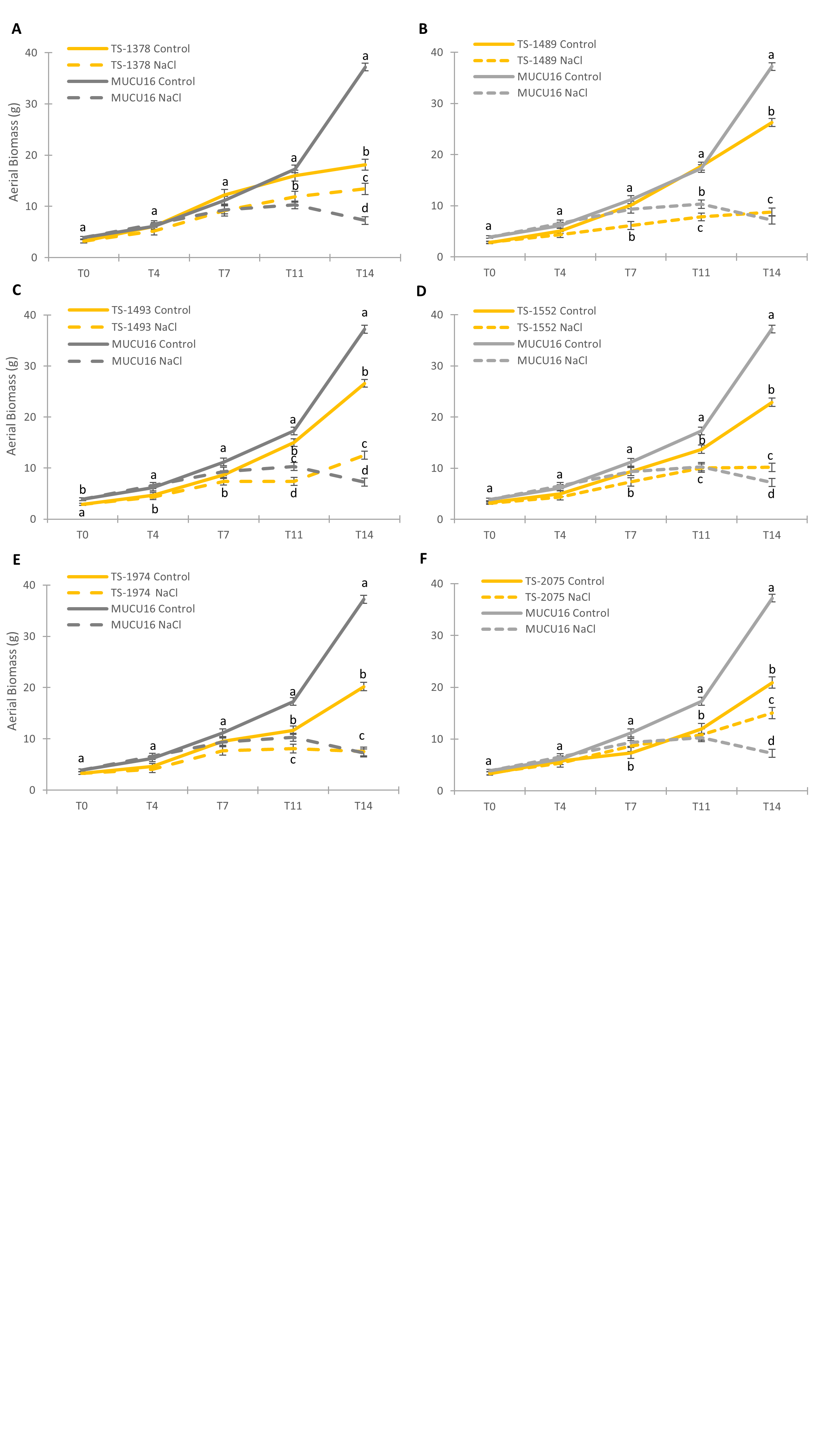

Supplement: Supplementary Figure 2 — Effect of salt stress on aerial biomass of MUCU16 and salt-tolerant mutant lines over time. (A-F) Comparison of the effect of salt stress on aerial biomass (g) of MUCU16 and each of the TS-mutant lines over time: T0 and T4, T7, T11, and T14 (4, 7, 11 and 14 days after starting the treatments). The results for each time point, genotype, and treatment come from the evaluation of 9–10 plants. The error bars represent SE. Different letters indicate statistically significant differences (p< 0.05) between samples for the same time period. [file Image2.tif]

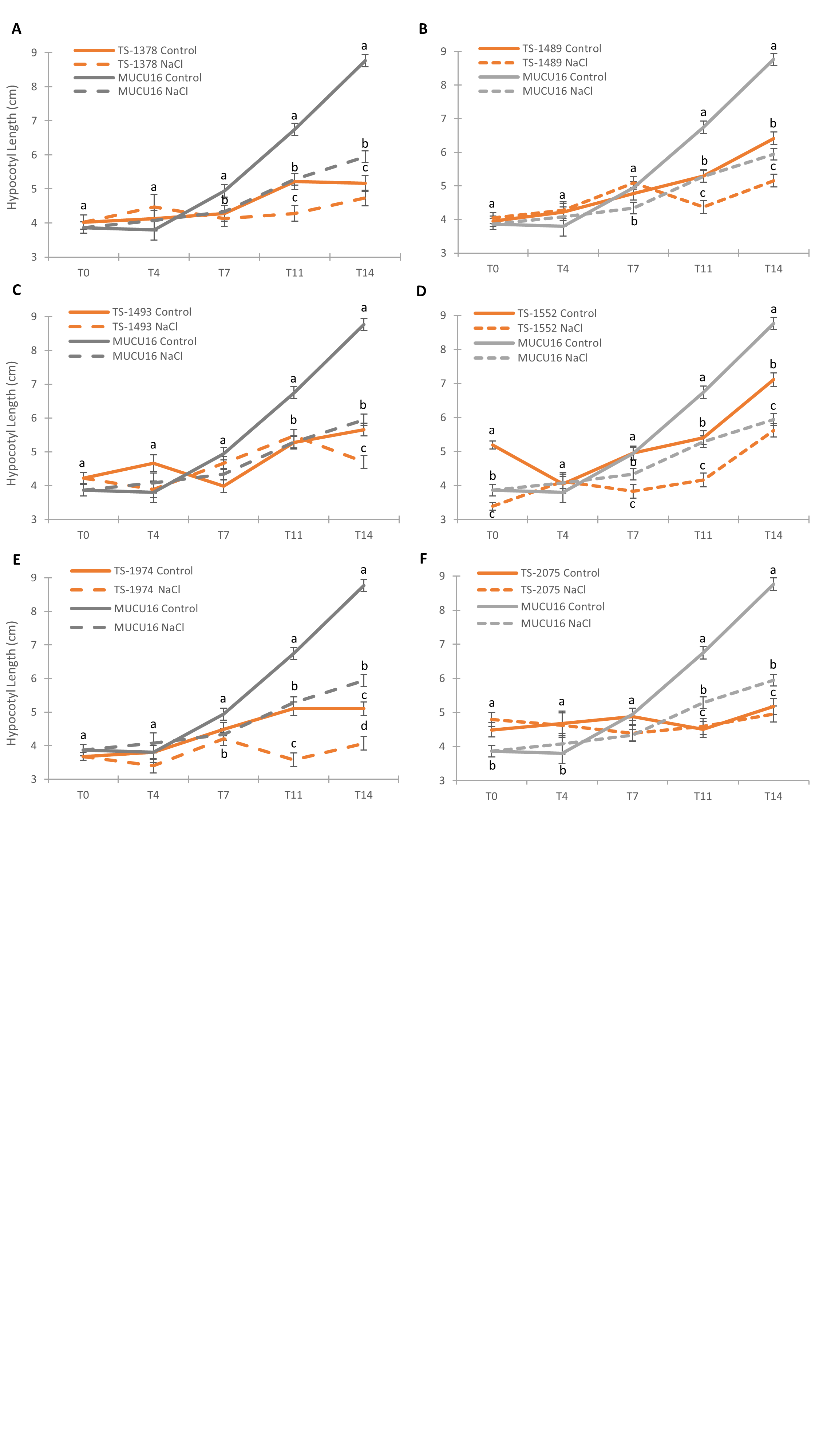

Supplement: Supplementary Figure 3 — Effect of salt stress on hypocotyl length of MUCU16 and salt-tolerant mutant lines over time. (A-F) Comparison of the effect of salt stress on the hypocotyl length (cm) of MUCU16 and each of the TS-mutant lines over time: T0 and T4, T7, T11, and T14 (4, 7, 11 and 14 days after starting treatments). The results for each time point, genotype, and treatment come from the evaluation of 9–10 plants. The error bars represent SE. Different letters indicate statistically significant differences (p< 0.05) between samples at the same time. [file Image3.tif]

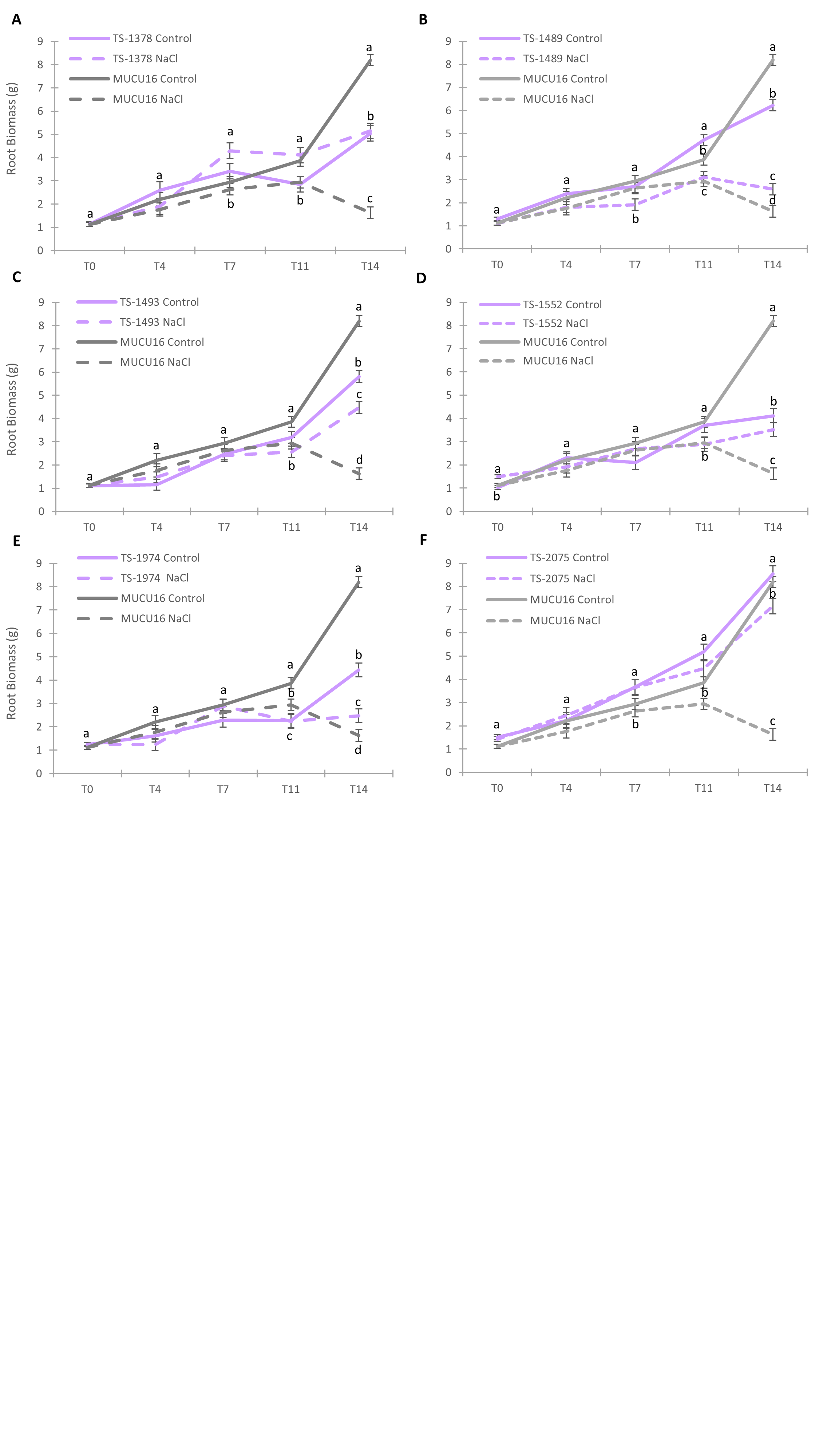

Supplement: Supplementary Figure 4 — Effect of salt stress on root biomass of MUCU16 and salt tolerant mutant lines over time. (A-F) Comparison of the effect of salt stress on root biomass (g) of MUCU16 and each of the TS-mutant lines over time: T0 and T4, T7, T11, and T14 (4, 7, 11 and 14 days after the beginning of the treatments). The results for each time point, genotype, and treatment come from the evaluation of 9–10 plants. The error bars represent SE. Different letters indicate statistically significant differences (p< 0.05) between samples at the same time. [file Image4.tif]

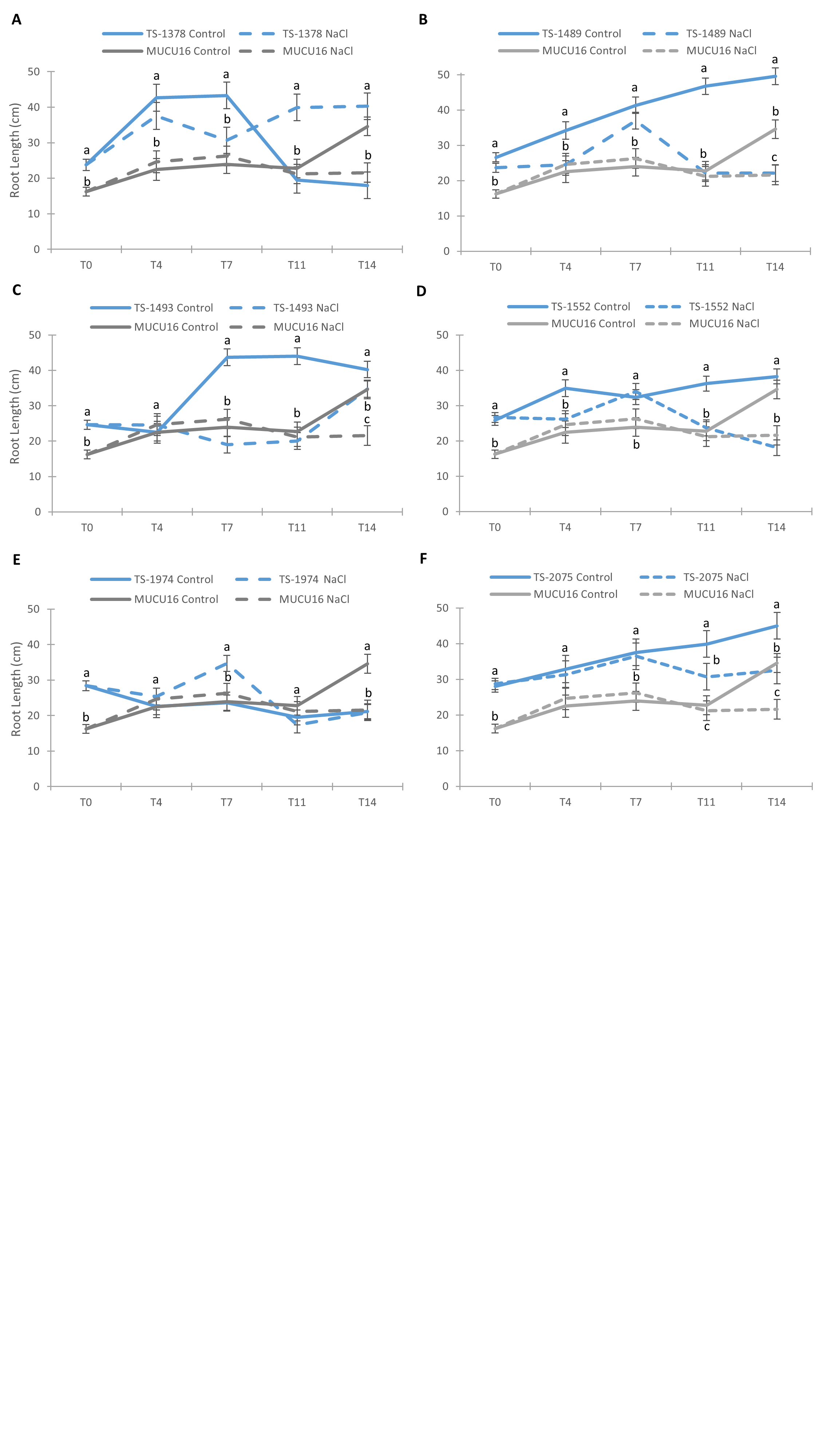

Supplement: Supplementary Figure 5 — Effect of salt stress on root length of MUCU16 and salt-tolerant mutant lines over time. (A-F) Comparison of the effect of salt stress on root length (cm) of MUCU16 and each of the TS-mutant lines over time: T0 and T4, T7, T11, and T14 (4, 7, 11 and 14 days after the beginning of the treatments). Results in each time point, genotype and treatment come from the evaluation of 9–10 plants. Error bars represent SE. Different letters indicate statistically significant differences (p< 0.05) between samples at the same time. [file Image5.tif]

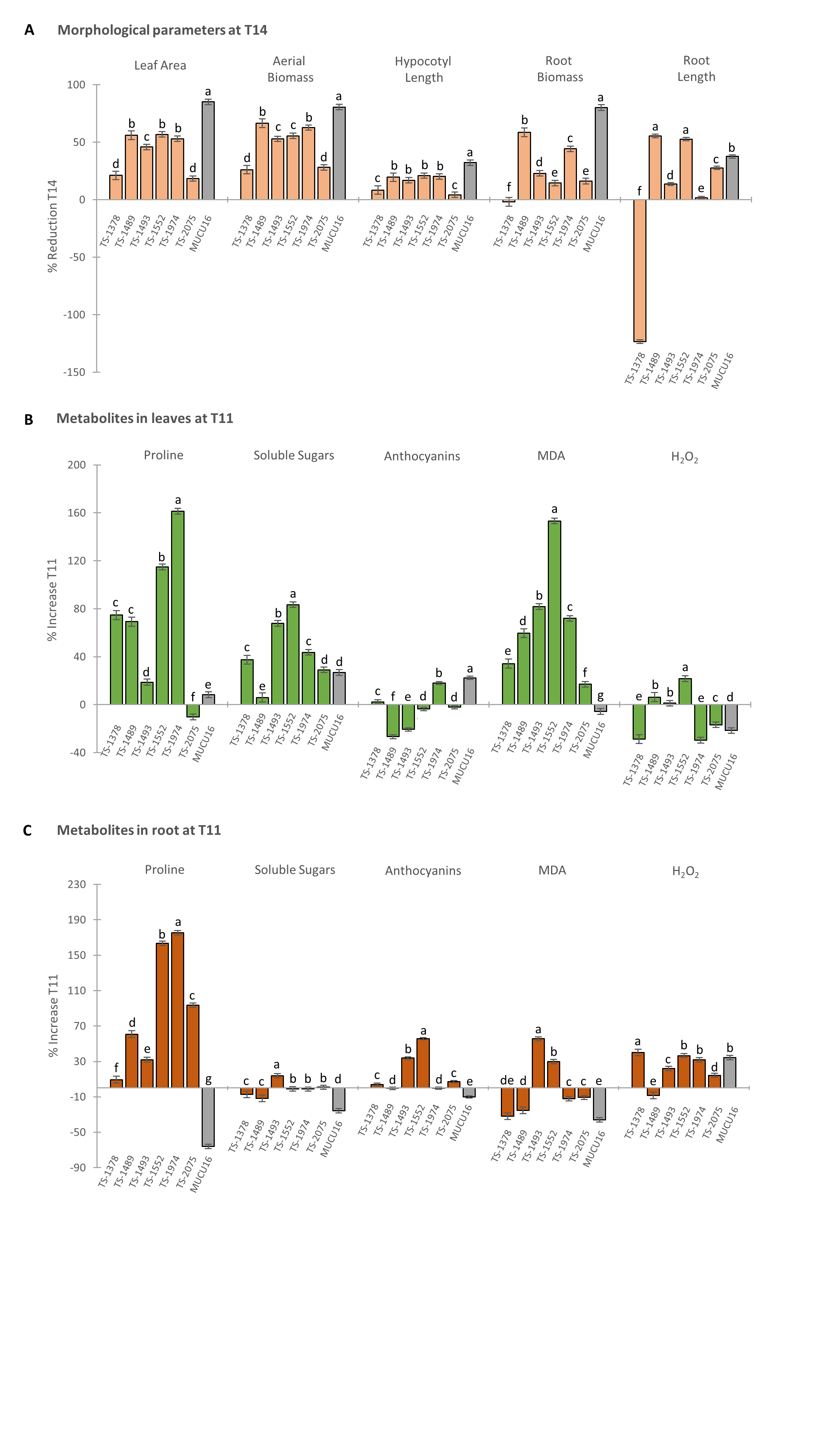

Supplement: Supplementary Figure 6 — Effect of salt stress on growth parameters and metabolites content of MUCU16 and salt-tolerant mutant plants. (A) Percentages of reduction at T14 (14 days after the beginning of the experiment) of each morphological parameter (root and hypocotyl length, aerial and root biomass, and leaf area) in response to salt stress. (B, C) Percentages of increase at T11 (11 days after the beginning of the experiment) of metabolite content (proline, soluble sugars, anthocyanins, MDA and H2O2) in response to salt stress of MUCU16 and mutant (B) leaves (dark green) and (C) root (dark brown). The genotype MUCU16 is always represented in gray. The percentages were calculated with respect to plants of the same genotype growing under control conditions. The error bars represent SE. Different letters indicate statistically significant differences (p< 0.05) between lines for the same parameter/metabolite. [file Image6.tif]
